# Rewiring of the N-Glycome with prostate cancer progression and therapy resistance

**DOI:** 10.1038/s41698-023-00363-2

**Published:** 2023-02-24

**Authors:** William Butler, Colin McDowell, Qing Yang, Yiping He, Yue Zhao, J. Spencer Hauck, Yinglu Zhou, Hong Zhang, Andrew J. Armstrong, Daniel J. George, Richard Drake, Jiaoti Huang

**Affiliations:** 1 Department of Pathology, Duke University School of Medicine, Durham, NC 27710 USA; 2 Department of Cell and Molecular Pharmacology and Experimental Therapeutics, Medical University of South Carolina, Charleston, SC 29425 USA; 3 School of Nursing, Duke University School of Medicine, Durham, NC 27710 USA; 4 Duke Cancer Institute, Duke University, Durham, USA; 5 Department of Pathology, College of Basic Medical Sciences and the First Hospital of China Medical University, Shenyang, China; 6 Department of Data Science, Dana-Farber Cancer Institute, Boston, MA 02215 USA; 7 Duke Cancer Institute Center for Prostate and Urologic Cancers, Department of Medicine, Division of Medical Oncology and Urology, Duke University School of Medicine, Durham, NC 27710 USA

**Keywords:** Prostate cancer, Glycobiology

## Abstract

An understanding of the molecular features associated with prostate cancer progression (PCa) and resistance to hormonal therapy is crucial for the identification of new targets that can be utilized to treat advanced disease and prolong patient survival. The glycome, which encompasses all sugar polymers (glycans) synthesized by cells, has remained relatively unexplored in the context of advanced PCa despite the fact that glycans have great potential value as biomarkers and therapeutic targets due to their high density on the cell surface. Using imaging mass spectrometry (IMS), we profiled the N-linked glycans in tumor tissue derived from 131 patients representing the major disease states of PCa to identify glycosylation changes associated with loss of tumor cell differentiation, disease remission, therapy resistance and disease recurrence, as well as neuroendocrine (NE) differentiation which is a major mechanism for therapy failure. Our results indicate significant changes to the glycosylation patterns in various stages of PCa, notably a decrease in tri- and tetraantennary glycans correlating with disease remission, a subsequent increase in these structures with the transition to therapy-resistant PCa, and downregulation of complex N-glycans correlating with NE differentiation. Furthermore, both nonglucosylated and monoglucosylated mannose 9 demonstrate aberrant upregulation in therapy-resistant PCa which may be useful therapeutic targets as these structures are not normally presented in healthy tissue. Our findings characterize changes to the tumor glycome that occur with hormonal therapy and the development of castration-resistant PCa (CRPC), identifying several glycan markers and signatures which may be useful for diagnostic or therapeutic purposes.

## Introduction

Prostate Cancer (PCa) is the most common non-cutaneous malignancy and a leading cause of cancer-related mortality in men over the age of 50^1^. Early stage, organ confined PCa has a high cure rate but some patients will experience disease recurrence after radical treatment^2^ which requires hormonal therapy to inhibit androgen receptor (AR) activity^3,4^. AR-targeted treatment is initially effective but inevitably leads to the development of castration resistant PCa (CRPC)^4^. Approximately 20% of CRPC is histologically classified as small cell neuroendocrine carcinoma (SCNC, also known as CRPC-NE) which is highly lethal^5^.

**Fig. 1:**
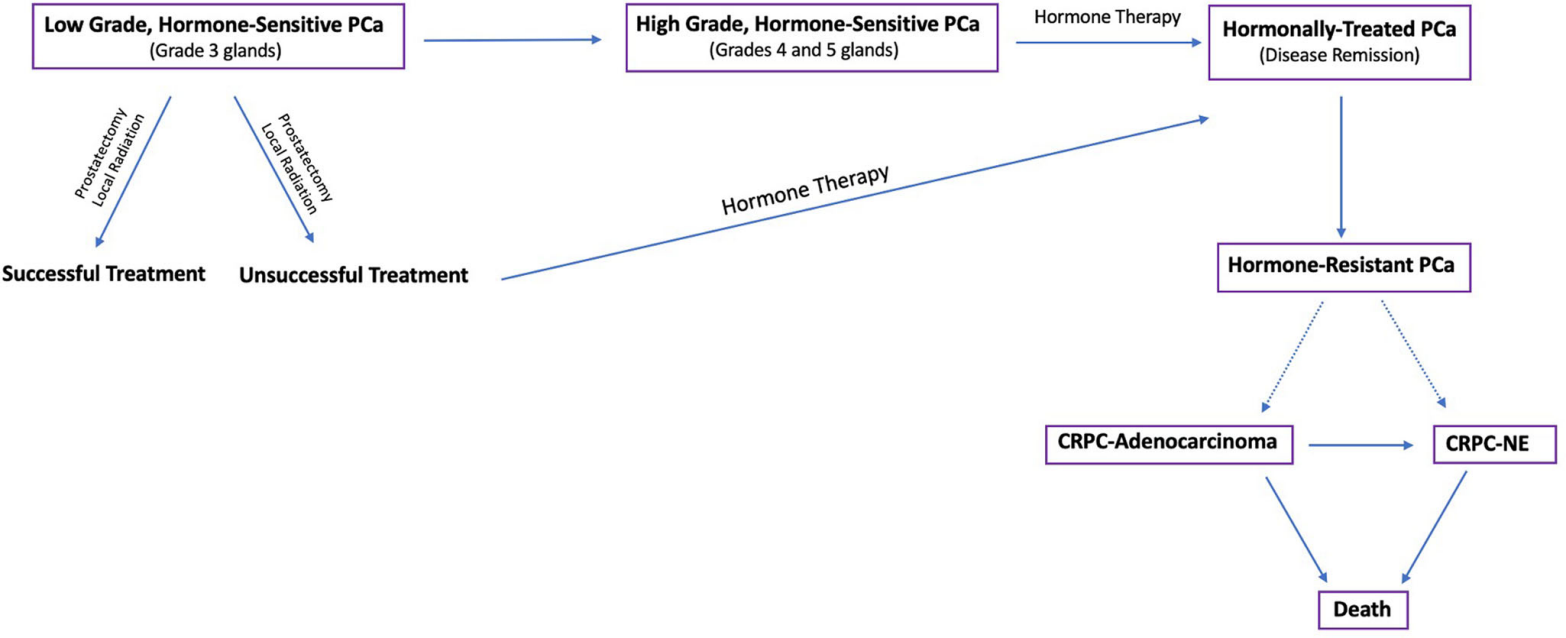
The Progression of Prostate Cancer. Flow diagram depicting the progression and treatment of PCa from low grade, hormone-sensitive PCa to late stage, hormone-resistant tumors.

Many studies have sought to profile the molecular changes associated with PCa progression, therapy resistance, and NE differentiation^5–9^, most of which have focused on changes in the genome, transcriptome, proteome, epigenome, and metabolome. In contrast, the glycome, which comprises all of the sugar polymers (glycans) synthesized by cells, has remained relatively unexplored in many cancer settings including PCa despite the fact that aberrant glycosylation is a well-known hallmark of cancer^10^ and the prostate is a major secretor of glycoproteins^11^. So far, studies on the PCa tumor glycome^12–14^ have only evaluated early stage disease or, in the setting of CRPC, changes in the serum glycome^15,16^, mostly because advanced PCa tissue is usually not available because the tissue is rarely biopsied or resected at this stage of the disease. Unfortunately, changes in the serum glycome are not necessarily tumor derived as systemic hormonal therapy has effects on many tissue types. Therefore, there remains an urgent, unmet need, to study changes to the PCa tumor glycome in order to better define the biology of disease progression (Fig. 1) and discover better diagnostic markers and therapeutic targets.

The development of N-glycan imaging mass spectrometry in 2013 (IMS)^17,18^ has provided great advance to the field of glycobiology^19–25^ and allows glycans to be profiled directly on formalin-fixed, paraffin-embedded (FFPE) tissue specimens from patients. This approach allows tumor regions to be annotated and glycans to be studied within these pure tumor regions without interfering signals from the surrounding stroma and benign tissue enabling the discovery of tumor-specific changes and molecular targets^17,26^. In this study, we used a combination of tissue microarray (TMA) and whole-slide cases to profile tumor regions derived from 131 patients, including hormone-sensitive PCa (*n* = 84 patients), hormonally-treated PCa (*n* = 37 patients), CRPC-adenocarcinoma (*n* = 5 patients), and CRPC-NE (*n* = 5 patients). In addition to studying glycosylation changes throughout the disease spectrum from early, hormone-sensitive to late, therapy-resistant tumors, we compared histologically heterogeneous tumors (low-grade vs. high-grade, adenocarcinoma vs. SCNC) to determine glycosylation changes associated with tumor heterogeneity within the same patients. Our studies seek to model the glycobiology of advanced, therapy-resistant PCa utilizing IMS for accomplishing such a task. In this process, we discovered novel molecular mechanisms and glycan biomarkers associated with commonly used hormonal therapy as well as therapy resistance, nominating potential therapeutic targets that may benefit patients who have exhausted currently available therapeutic options.

## Results

### Loss of Gal3Fuc2Man3GlcNAc6 (2361.8560 m/z) and Gal3Fuc1Neu5Ac1Man3GlcNAc6 (2528.8833 m/z) is associated with loss of gland differentiation in early stage PCa

In early-stage, hormone-sensitive PCa, the biologic behavior is determined by the degree of glandular differentiation, pathologically known as Gleason patterns (or Gleason grades)^27^. To determine glycosylation changes associated with tumor behavior, we surveyed four patients with hormone-sensitive adenocarcinoma that had discrete, well-circumscribed regions within each tumor representing either well-differentiated glands (Gleason pattern 3) or poorly-differentiated glands (Gleason patterns 4 and 5).

**Fig. 2:**
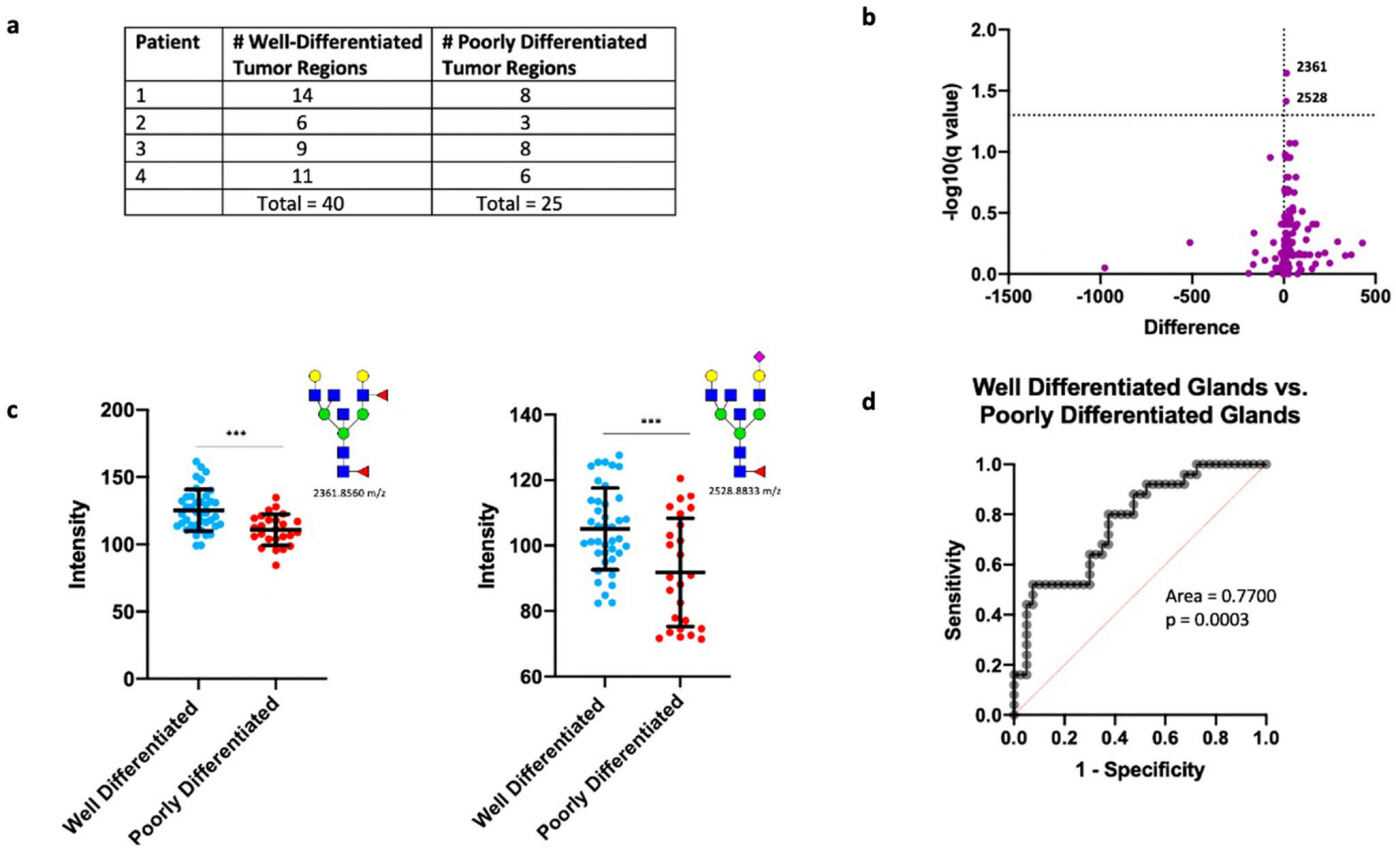
*N*-linked glycosylation changes associated with loss of differentiation. **a** Table demonstrating the number of annotated well-differentiated and poorly differentiated tumor regions for each patient. **b** Volcano plot comparison of all glycans in annotated well-differentiated tumor regions (*n* = 40) and annotated poorly differentiated tumor regions (*n* = 25) with 2361.8560 m/z and 2528.8833 m/z showing statistical significance between the two groups. **c** Scatter plot comparison of Gal3Fuc2Man3GlcNAc6 and Gal3Fuc1Neu5Ac1Man3GlcNAc6 between all well differentiated (*n* = 40) and poorly differentiated (*n* = 25) tumor regions. Statistics performed between each group by multiple, unpaired t test with *p* < 0.05 considered significant. The following system was used to denote significance: *p* < 0.05 (*), *p* < 0.01 (**), *p* < 0.0001 (***), *p* < 0.00001 (****). Error bars correspond to standard deviation. **d** Receiver operator characteristic (ROC) curve demonstrating the predictive power (AUC = 0.77, *p* = 0.0003) of Gal3Fuc2Man3GlcNAc6 and Gal3Fuc1Neu5Ac1Man3GlcNAc6 loss for identifying poor differentiation of glands.

Multiple regions from each tumor representing different tumor grades were selected for glycome profiling (Fig. 2a and Supplementary Fig. 2). We first performed a statistical comparison (see Methods) of all detected glycans between well-differentiated tumor regions (n = 40) and poorly-differentiated tumor regions (*n* = 25) across all patients. Interestingly, only 2/150 glycans detected by IMS (Gal3Fuc2Man3GlcNAc6 (2361.8560 m/z) and Gal3Fuc1Neu5Ac1Man3GlcNAc6 (2528.8833 m/z)) demonstrated a statistically significant change (Fig. 2b, c), with both structures downregulated in poorly-differentiated tumor regions. In order to account for correlation within each patient, we built a multi-level model (mixed effects model) with individual patients considered to be a random effect. Both 2361.8560 m/z and 2528.8833 m/z remained highly significant (*p* = 6.57 × 10^−5^ and 1.1 × 10^−5^, respectively) indicating that loss of these structures is associated with loss of gland differentiation in early stage PCa.

Both structures are triantennary glycans containing bisecting N-acetylglucosamine (GlcNAc) and core fucose, with 2361.8560 m/z containing an outer arm fucose and 2528.8844 m/z containing a terminal sialic acid. Importantly, their loss of abundance in combination predicted poorly-differentiated tumor regions (AUC = 0.7700) (Fig. 2d). The surprisingly low number of significantly changed glycans suggests that there is either a very minimal change in N-glycosylation with loss of gland differentiation or that there is a high degree of interpatient variability with these two glycans representing the most consistent change across all patients studied. Indeed, intratumoral analysis of each patient tumor demonstrated a high degree of variability. Both patients 1 and 4 demonstrated minimal change detectable by T-test with Patient 1 demonstrating a high degree of upregulation of two hybrid structures (1136.3964 m/z and 1282.4543 m/z) and a core fucosylated paucimannose structure (1079.3749 m/z) (Supplementary Fig. 3). Alternatively, Patients 2 and 3 exhibited highly significant change between well-differentiated and poorly-differentiated tumor regions. Patient 2 demonstrated 25/150 glycans showing significant change, with 24 structures upregulated and 1 hybrid structure (1460.5020 m/z) downregulated (Supplementary Fig. 3) in poorly-differentiated regions. In this patient, the vast majority of glycans upregulated were tri- and tetraantennary glycans containing core fucose, with the majority of upregulated triantennary glycans also containing bisecting N-acetylglucosamine (GlcNAc) (Supplementary Fig. 3). In Patient 3, 83/150 glycans demonstrated significant change, with 30 structures upregulated and 53 structures downregulated in poorly-differentiated tumor regions (Supplementary Fig. 3). Upregulated glycans consisted mostly of hybrid and complex type glycans, with a higher proportion of biantennary structures (Supplementary Fig. 3). Downregulated glycans were mostly tri- and tetraantennary structures, with most triantennary glycans containing bisecting GlcNAc, an opposite trend to what was observed with patient 2 (Supplementary Fig. 3).

In conclusion, both Gal3Fuc2Man3GlcNAc6 (2361.8560 m/z) and Gal3Fuc1Neu5Ac1Man3GlcNAc6 (2528.8833 m/z) demonstrate consistently lower abundance in poorly-differentiated tumor regions across all patients tested. This experiment also demonstrates a high degree of interpatient variability when comparing glycans associated with loss of gland differentiation, both in regards to the amount of glycosylation affected and the specific classes of structures affected.

### Hormonal therapy induces significant upregulation of complex biantennary structures and downregulation of hybrid and complex tri- and tetraantennary structures

**Fig. 3:**
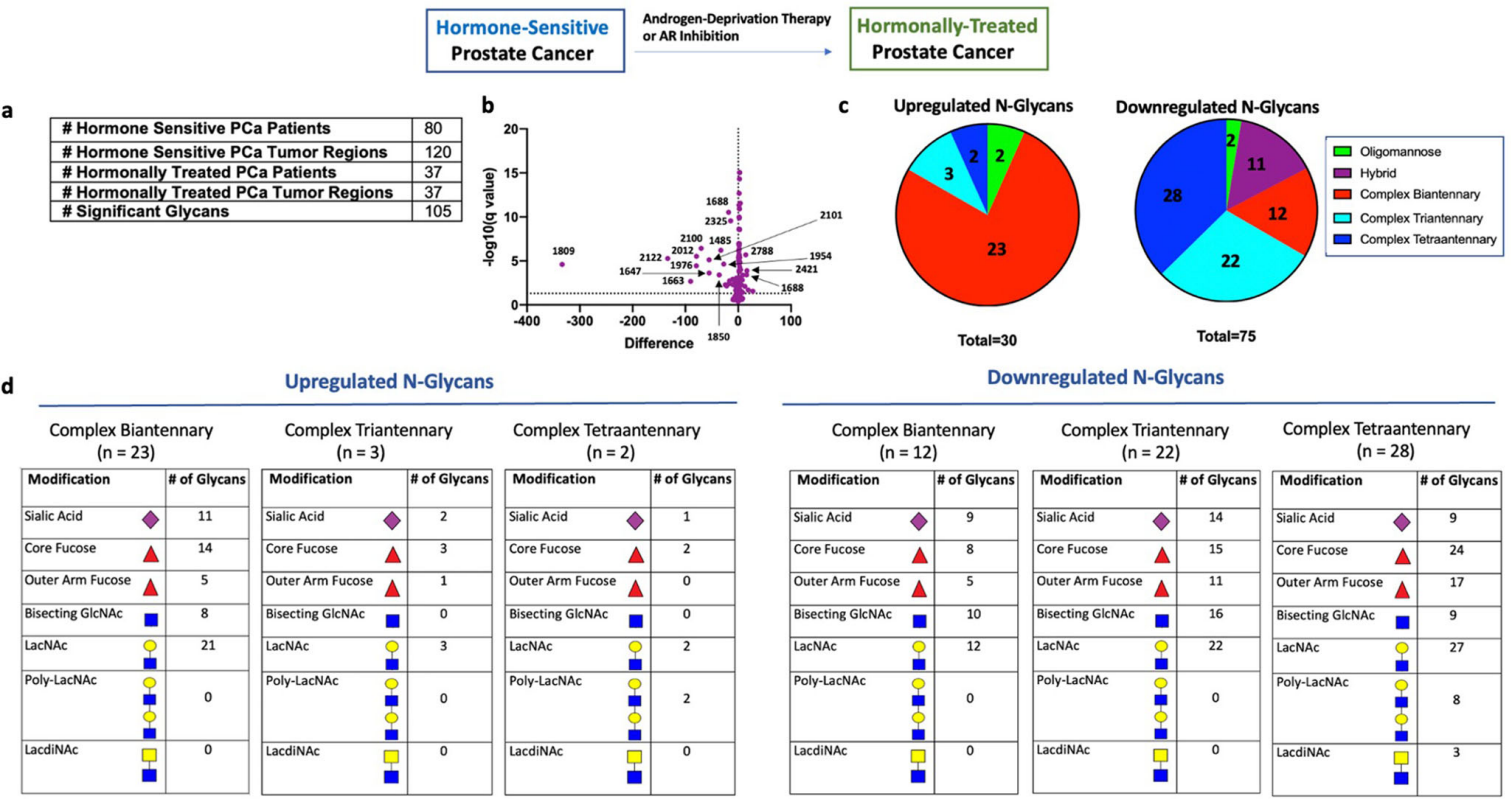
N-linked glycosylation changes in hormonally-treated tumors. **a** Table demonstrating number of hormone-sensitive and hormonally-treated PCa patients studied, the number of annotated tumor regions within each group, and number of significant glycans between all hormone-sensitive regions (*n* = 120) and all hormonally-treated regions (*n* = 37). **b** Volcano plot comparison of all glycans in annotated hormone sensitive PCa tumor regions (*n* = 120) and annotated hormonally-treated PCa tumor regions (*n* = 37). 105/150 glycans detected demonstrate statistical significance between the two groups with 30 structures upregulated and 75 structures downregulated. **c** Circle graph demonstrating the proportion of upregulated and downregulated glycans within each major structural category. Complex biantennary glycans represent the largest proportion of upregulated glycans while complex tri- and tetraantennary glycans represent the largest proportion of downregulated glycans. **d** Tables demonstrating common structural modifications associated with upregulated and downregulated *N*-glycans in each major structural class showing a significant change.

Hormonal therapy to inhibit AR activity has been the main modality to treat advanced and metastatic PCa^2^. In order to determine changes in glycosylation associated with AR inhibition but not yet in the state of hormonal resistance, we compared the tumor regions derived from patients with hormone sensitive PCa (*n* = 80 patients) and patients with hormonally-treated PCa (*n* = 37 patients) (Fig. 3a). All hormonally-treated PCa tissues were from patients who received neoadjuvant hormonal therapy (leuprolide acetate) for 3–6 months before prostatectomy, representing hormonally-treated tumors that are not yet castration-resistant.

Statistical comparison of all hormone-sensitive PCa regions (*n* = 120) with all hormonally-treated PCa regions (*n* = 37) (Fig. 3a) demonstrated 105/150 glycans showing a significant change in hormonally-treated tumors, with 30 structures upregulated and 75 structures downregulated (Fig. 3b, c). Upregulated glycans were predominately complex biantennary structures (Fig. 3c, d). Core fucose and sialic acid were common modifications to these biantennary structures while bisecting GlcNAc and outer arm fucose were less common (Fig. 3d). Interestingly, in addition to these structures being increased within the annotated tumor regions, they are also increased in the surrounding stroma indicating that many of these glycans may be also be found on secreted proteins (Supplementary Fig. 4). In contrast, downregulated glycans were primarily complex tri- and tetraantennary structures (Fig. 3c, d and Supplementary Fig. 5). Hybrid and complex biantennary glycans containing bisecting GlcNAc also demonstrated significant downregulation (Fig. 3c, d). Core fucose and sialic acid were common modifications to all complex glycans showing significant change (Fig. 3d). The majority of downregulated triantennary structures (16/22) contained bisecting GlcNAc (Fig. 3d). Of the 3 triantennary structures upregulated in hormonally-treated PCa, none contained bisecting GlcNAc (Fig. 3d). Furthermore, several structures in hormonally-treated tissue showed a high magnitude of change suggesting that they may be good markers of sensitivity to hormonal manipulation (Fig. 3b and Supplementary Fig. 6).

These results indicate that hormonal therapy induces upregulation of complex biantennary structures and downregulation of complex tri- and tetraantennary structures. Hybrid structures and complex biantennary structures containing bisecting GlcNAc were also downregulated in hormonally-treated tumors. Core fucose and sialic acid are common modifications to all involved complex glycans, while bisecting GlcNAc was significantly less common in hormonally-treated tumors.

### Resistance to hormonal therapy is associated with upregulation of complex tri- and tetraantennary glycans

**Fig. 4:**
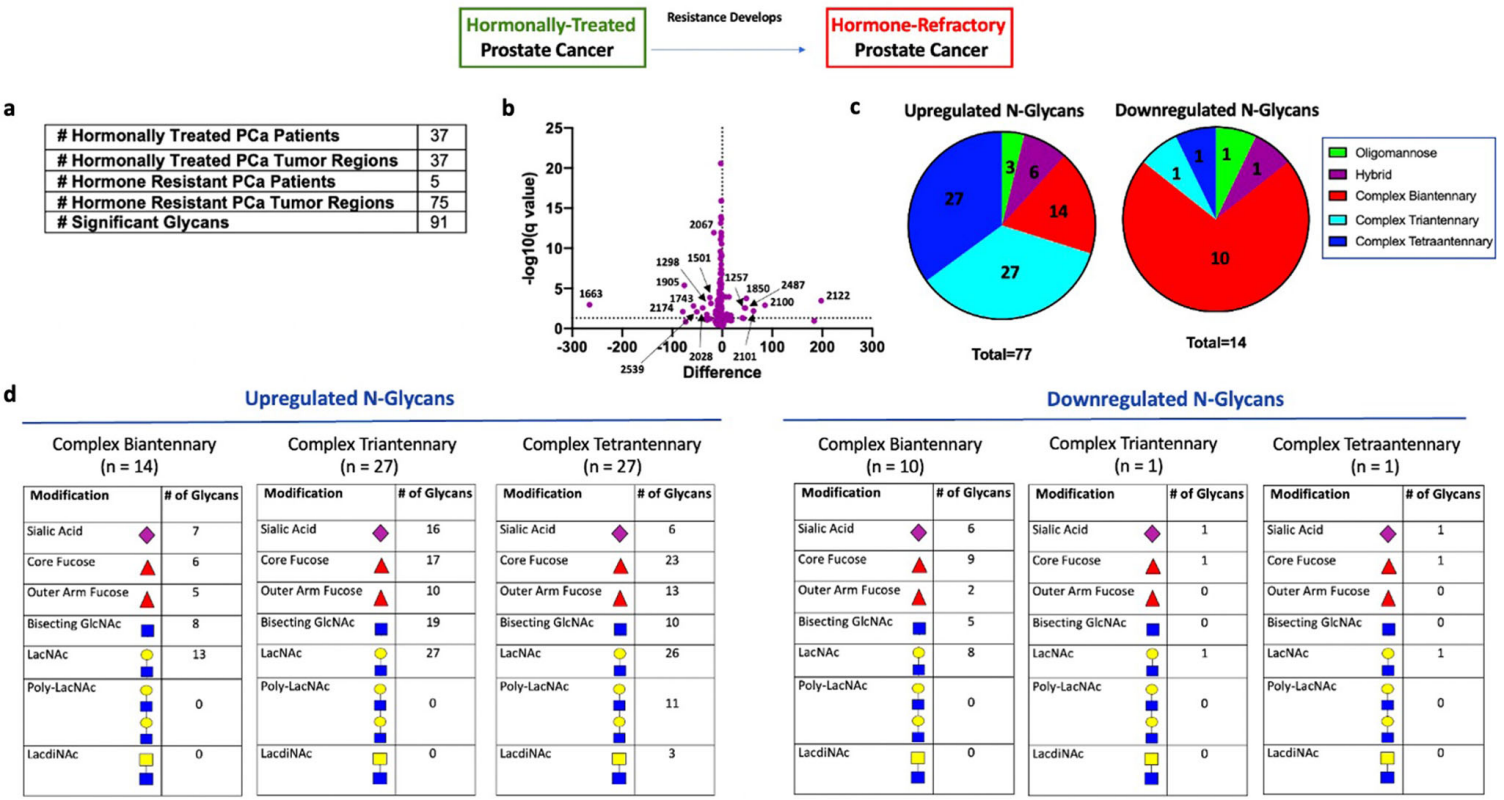
N-linked glycosylation changes in hormone resistant tumors. **a** Table demonstrating the number of hormonally treated and hormone resistant PCa patients studied, the number of annotated tumor regions within each group, and number of significant glycans between all hormonally treated tumor regions (*n* = 37) and all hormone resistant tumor regions (*n* = 75). **b** Volcano plot comparison of all glycans in annotated hormonally treated PCa tumor regions (*n* = 37) and annotated hormone resistant PCa tumor regions (*n* = 75). 91/150 glycans detected demonstrate statistical significance between the two groups with 77 structures upregulated and 14 structures downregulated. **c** Circle graph demonstrating the proportion of upregulated and downregulated glycans within each major structural category. Complex tri- and tetraantennary glycans represent the largest proportion of upregulated glycans while complex biantennary glycans represent the largest proportion of downregulated glycans. **d** Tables demonstrating common structural modifications associated with upregulated and downregulated *N*-glycans in each major structural class showing a significant change.

Despite initial activity, resistance to hormonal therapy is inevitable and an understanding of the molecular features associated with therapy resistance is critical to finding new therapeutic targets^2^. To determine glycosylation changes associated with therapy resistance, we compared the tumor regions from patients with hormonally-treated PCa (*n* = 37) and patients with hormone-resistant PCa (*n* = 5) (Fig. 4a).

Statistical comparison of all hormonally-treated PCa regions (*n* = 37) and all hormone-resistant PCa regions (*n* = 75) (Fig. 4a) demonstrated 91/105 glycans showing a statistically significant change in hormone-resistant tumors, with 77 structures upregulated and 14 structures downregulated (Fig. 4b, c). Upregulated structures were predominately complex tri- and tetraantennary structures (Fig. 4c, d). Bisecting GlcNAc was commonly found in upregulated triantennary glycans (19/27) and sialic acid was rare in upregulated tetraantennary glycans (6/27) (Fig. 4d). Core fucose was commonly found on all upregulated complex tri- and tetraantennary glycans (Fig. 4d). In contrast, downregulated structures were predominately complex biantennary structures, most containing core fucose and sialic acid (Fig. 4d).

We then studied PCa tumor models to confirm the aberrant upregulation of complex tri- and tetraantennary structures in CRPC. Here, we surveyed three hormone-sensitive PCa cell lines (LNCaP, VCaP, and LAPC4) and three castration-resistant lines (C42, CS2, and PC3) for phytohemaggluttin-L (PHA-L) reactivity, which correlates with the abundance of β1-6 complex tri- and tetraantennary N-glycans. Here, we observe low levels in hormone-sensitive PCa lines (LNCaP: 6.26%, VCaP: 3.09%, LAPC4: 6.19%) with significantly higher levels in hormone-resistant PCa lines (C42: 14.0%, CS2: 11.9%, PC3: 13.4%) (Supplementary Figs. 7, 8). We further show that treatment of LNCaP cells with androgen deprivation for 7–14 days, which models the development of CRPC, induced the formation of these complex tri- and tetraantennary structures as well as an increase in several of the enzymes involved in this pathway (Supplementary Fig. 9).

**Fig. 5:**
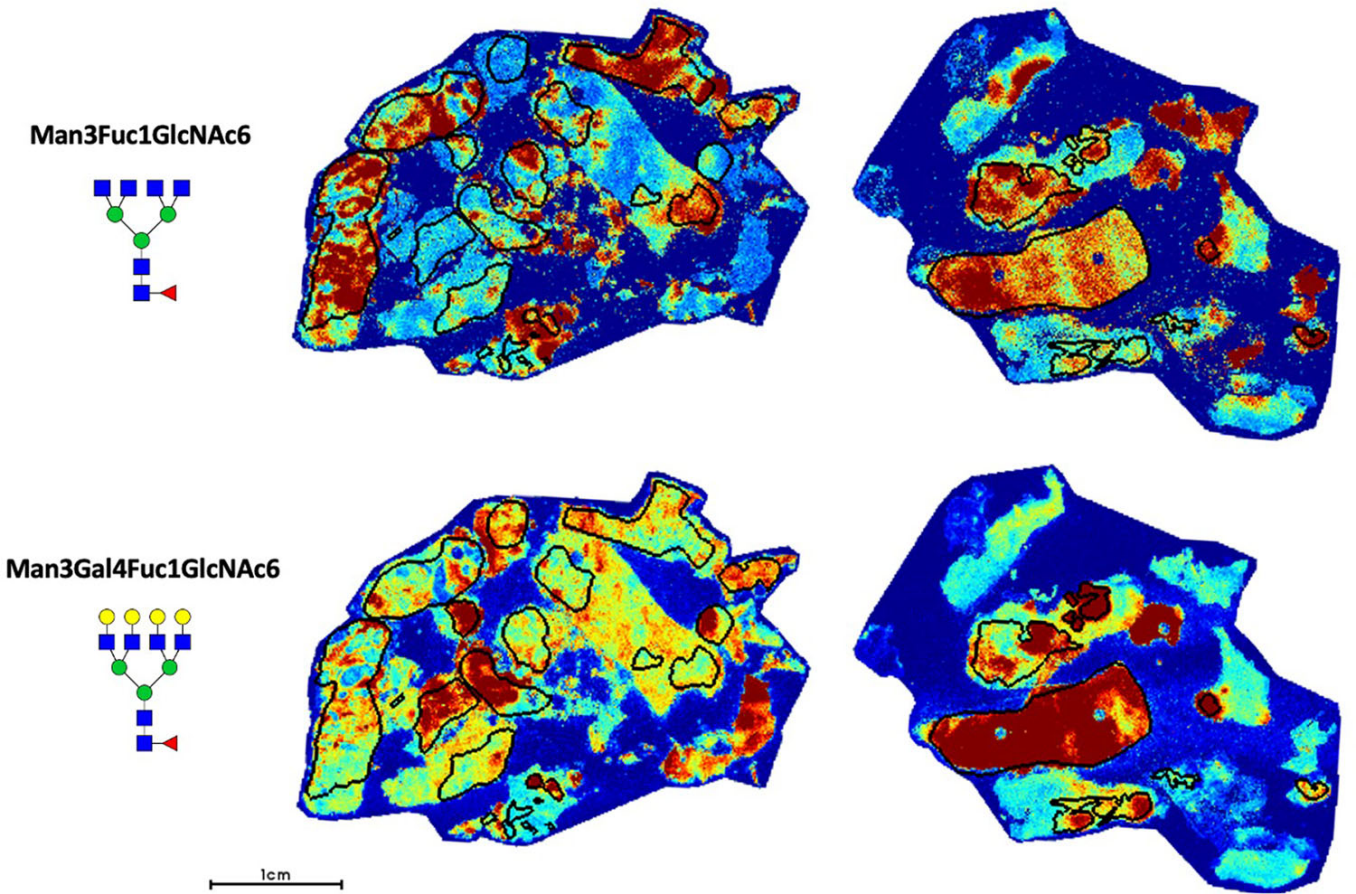
Representative glycan image demonstrating Man3Fuc1GlcNAc6 (1891.6924 m/z) and Man3Gal4Fuc1GlcNAc6 (2539.9037 m/z) localization within demarcated tumor regions (black circles) in two hormone resistant PCa cases.

These results suggest that increased branching from complex biantennary to complex tri- and tetraantennary forms is an important feature associated with the development of hormone therapy resistance. In considering whole tissue, these resulting glycans are most commonly abundant within the annotated tumor regions with significantly lower signal in surrounding tissue (Fig. 5). Interestingly, publicly available data^28^ demonstrates that several enzymes involved in the synthesis of these multiantennary structures, particularly MGAT4B, MGAT5, and MGAT5B, show aberrant upregulation in CRPC compared to hormone-sensitive PCa (Supplementary Fig. 10) suggesting increased expression of these enzymes might contribute to this observed phenotype. In our data, the majority of upregulated complex triantennary glycans contain bisecting GlcNAc (Fig. 4d). As these structures were found to be downregulated following hormonal therapy, triantennary structures containing bisecting GlcNAc appear correlated with disease activity. Most upregulated tetraantennary glycans are non-sialylated (Fig. 4d) which may be a mechanism to allow increased binding to galectins, although this requires further study.

### Mannose 8, mannose 9, and glucosylated mannose 9 are associated with CRPC

High mannose structures are commonly found on tumor cells^26^. In healthy cells, these glycans are usually not found in high abundance as they are normally processed to complex forms by the action of various glycosidases and glycosyltransferases^29^. Consequently, their presence on the cell surface has been shown to induce autoantibody response^30^ indicating these structures are immunogenic and have the potential to be good therapeutic targets.

**Fig. 6:**
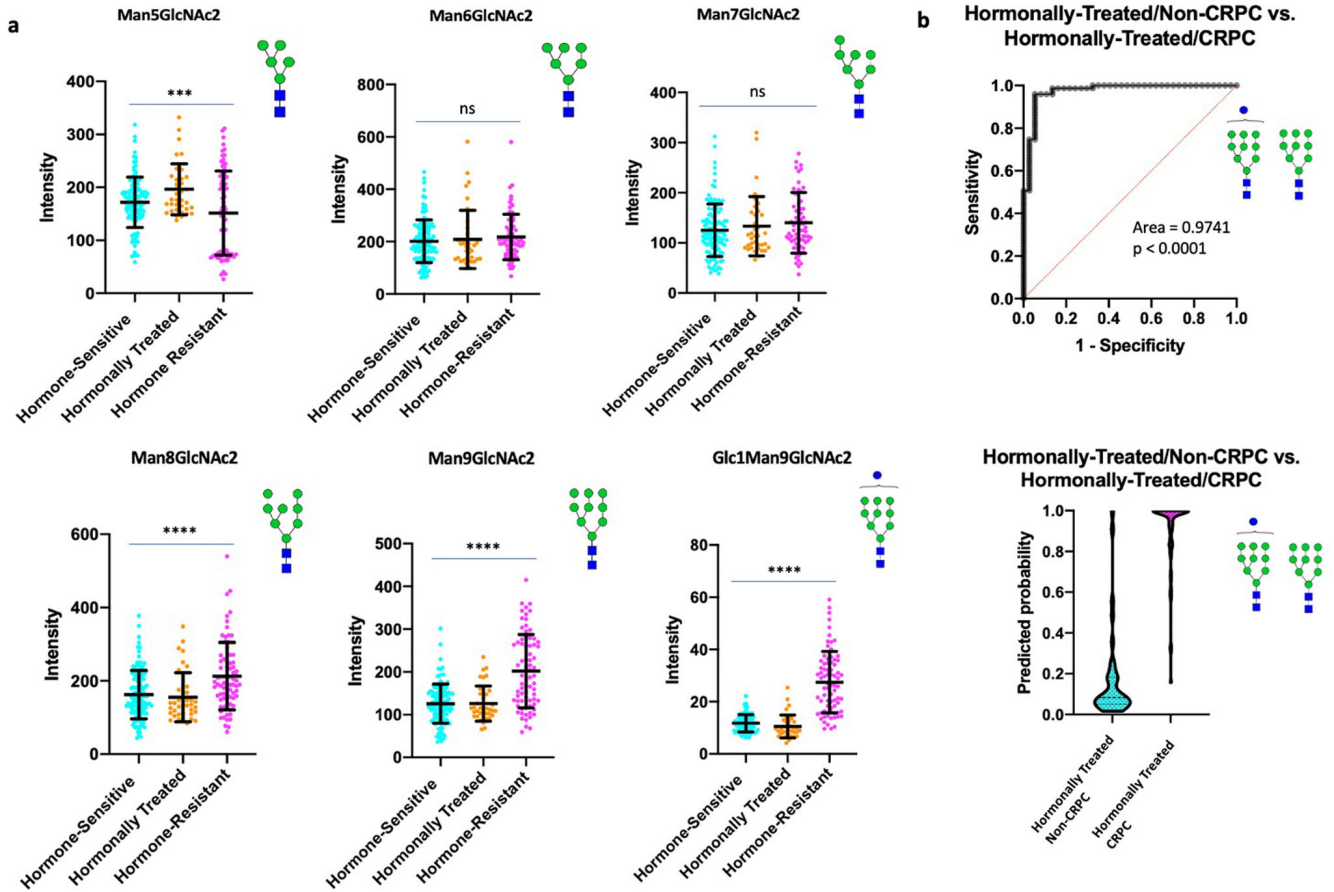
High mannose structures associated with resistance to hormonal therapy. **a** Scatter plot comparisons of Man5–9 abundance in hormone-sensitive (*n* = 120), hormonally-treated (*n* = 37), and hormone-resistant (*n* = 75) tumor regions. One-way ANOVA was used for multiple comparisons with *p* < 0.05 considered significant. The following system was used to denote significance: *p* < 0.05 (*), *p* < 0.01 (**), *p* < 0.0001 (***), *p* < 0.00001 (****). Error bars correspond to standard deviation. **b** (Top) ROC curve demonstrating the predictive power (AUC = 0.9741, *p* < 0.0001) of Man9 and Glc1Man9 for disease recurrence (Bottom) Violin plot demonstrating the probability of Man9 and Glc1Man9 for predicting disease recurrence (Center line represents median, Upper and lower bars represent 75th and 25th quartiles, respectively).

**Fig. 7:**
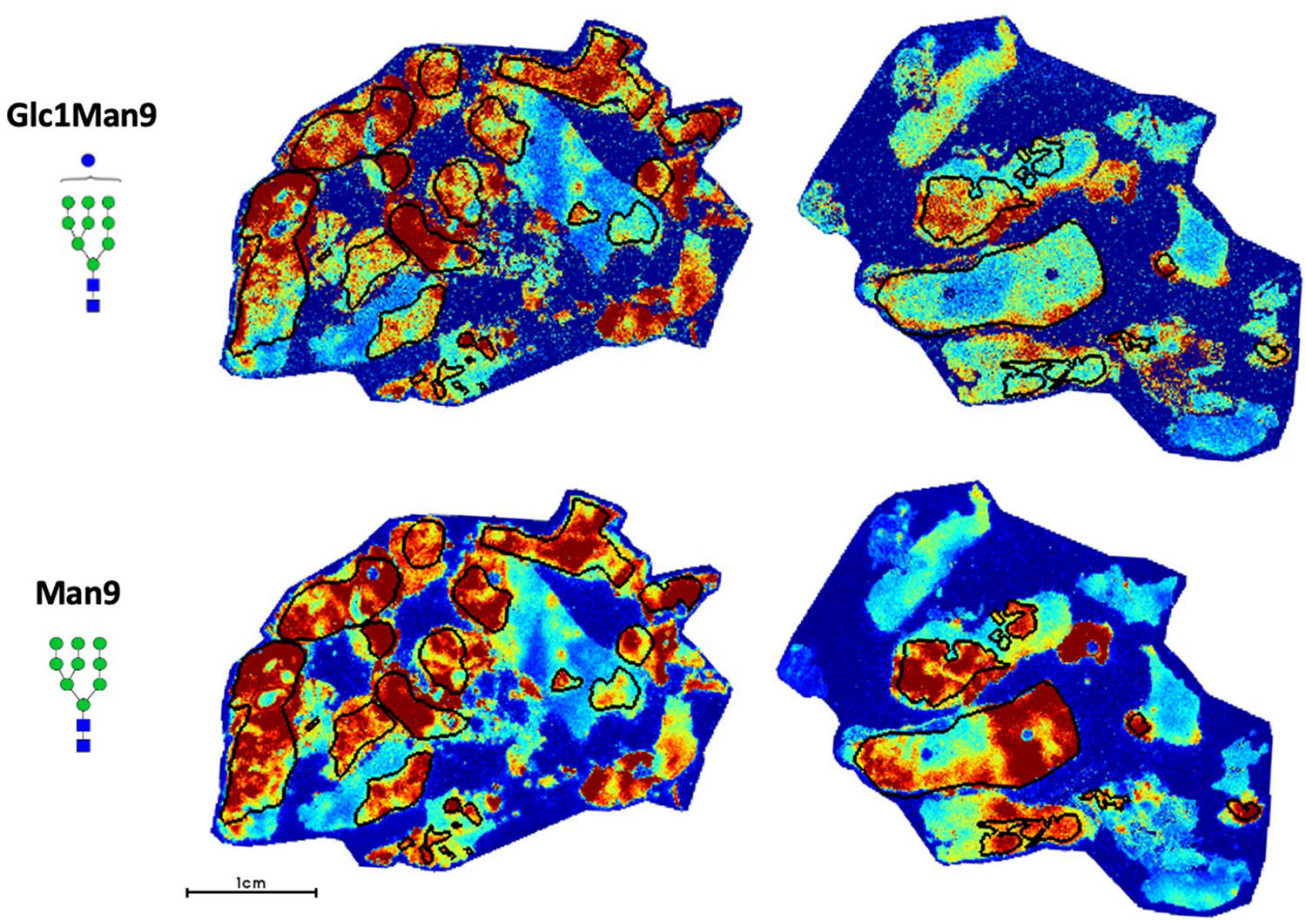
Representative glycan image demonstrating Glc1Man9 and Man9 localization within demarcated tumor regions (black circles) in two hormone resistant PCa cases. Both glycans demonstrate high specificity for tumor regions.

In our study, we observe a transition from lower molecular weight high mannose structures (Man5–7) to higher molecular weight high mannose structures (Man8–9) in hormone-resistant PCa (Fig. 6a). In particular, Man9 and Glc1Man9 demonstrate the highest specificity for hormone-resistant tumors (Fig. 6a, b) with an AUC = 0.9741. In addition to being highly upregulated in hormone-resistant PCa, they demonstrate a high degree of colocalization within the tumor (Fig. 7). Given that these structures are less-processed than lower molecular weight high mannose forms, they are likely to be more immunogenic.

Man8, Man9, and Glc1Man9 were among the 9 upregulated glycans showing the highest magnitude of change in hormone-resistant tumors, suggesting good diagnostic sensitivity for CRPC (Supplementary Fig. 11). As these structures also have high tumor specificity, it would be important to explore whether these glycans can serve as markers for tumor imaging, radioligand therapies, circulating tumor cells (CTCs) or targets for immunological based therapies in future work.

### Neuroendocrine differentiation is associated with downregulation of complex N-glycans

**Fig. 8:**
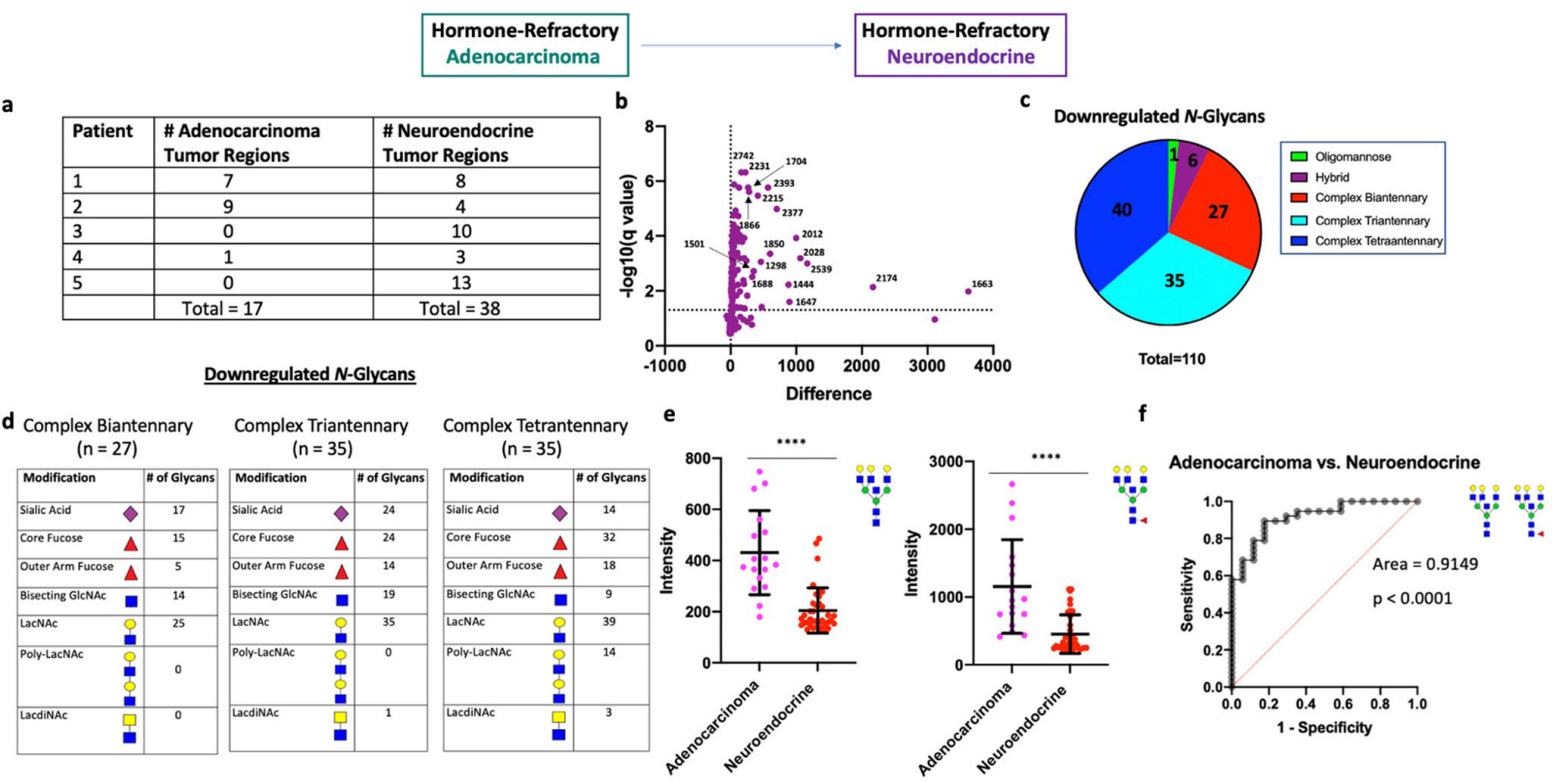
N-linked glycosylation changes associated with neuroendocrine differentiation. **a** Table demonstrating number of annotated adenocarcinoma and NE tumor regions for each patient. **b** Volcano plot comparison of all glycans in annotated adenocarcinoma regions (*n* = 17) and annotated NE tumor regions (*n* = 38). 110/150 glycans detected demonstrate statistical significance between the two groups with all structures downregulated. **c** Circle graph demonstrating the proportion of downregulated glycans within each major structural category. The vast majority of downregulated glycans were complex-type with roughly equal proportions of complex bi-, tri-, and tetraantennary structures. **d** Table demonstrating common structural modifications associated with the downregulated *N*-glycans in each major structural class showing a significant change. **e** Scatter plot comparison of fucosylated and non-fucosylated Gal3Man3GlcNAc6 between all adenocarcinoma (*n* = 17) and NE (*n* = 38) tumor regions. Statistics performed between each group by multiple, unpaired t test with *p* < 0.05 considered significant. The following system was used to denote significance: *p* < 0.05 (*), *p* < 0.01 (**), *p* < 0.0001 (***), *p* < 0.00001 (****). Error bars correspond to standard deviation. **f** ROC curve demonstrating the predictive power (AUC = 0.9149, *p* < 0.0001) of fucosylated and non-fucosylated Gal3Man3GlcNAc6 for predicting NE differentiation.

At disease onset, >99% of PCa is histologically classified as adenocarcinoma which consists of AR + tumor cells forming glandular structures which respond to hormonal therapy, albeit temporarily^31^. In metastatic, castration-resistant PCa, ~17–20% are histologically classified as SCNC^5^, consisting of tumor cells that show NE differentiation and are completely unresponsive to hormonal therapy due to inactivation of AR signaling. Although their origin remains debatable, evidence more strongly supports that NE cells in SCNC arise from adenocarcinoma tumor cells through a process called lineage plasticity^32^. A better understanding of the molecular features associated with SCNC is critical for developing novel therapies to treat these currently untreatable tumors. We studied 5 patients with SCNC (CRPC-NE), among which 3 had SCNC regions co-existing with adenocarcinoma and 2 had pure SCNC histology (Fig. 8a and Supplementary Fig. 12). This allowed us to compare mixed adenocarcinoma and NE regions to study glycosylation changes associated with SCNC transdifferentiation.

We subsequently performed a statistical comparison of all mixed adenocarcinoma tumor regions (*n* = 17) and all SCNC regions (*n* = 38) across all patients. Here, 110/150 glycans showed significant quantitative change with all significant glycans being downregulated (Fig. 8b, c). Interestingly, 102/110 were complex N-glycans and included roughly similar proportions of biantennary, triantennary, and tetraantennary forms (Fig. 8c, d). The levels of high mannose glycans were largely consistent with CRPC-adenocarcinoma regions with only Glc1Man9 demonstrating slight, but significant, downregulation (Supplementary Fig. 13). As Glc1Man9 is a key component of the unfolded protein response (UPR)^33^, this suggests high mannose forms are largely unaffected with NE differentiation but there might be slightly lower UPR activity in NE cells compared to adenocarcinoma tumor cells.

Since one patient in the analysis with mixed SCNC (Patient 1) had a high number of tumor regions containing roughly equal proportions of adenocarcinoma and NE histology (Fig. 8a), we were able to perform intratumoral analysis for this patient which demonstrates significant downregulation of complex N-glycans in the NE tumor regions (Supplementary Fig. 14), consistent with the overall analysis. Although patient 2 also had a heterogeneous tumor with >2 regions per histologic category, we were unable to capture significant differences between the two histological regions which we reason is due to smaller magnitude changes coupled with lower statistical power. Alternatively, it is possible that the NE cells in this patient are less differentiated from the adenocarcinoma tumor cells at the time point this tissue was resected as this patient had a higher proportion of adenocarcinoma.

Both non-fucosylated and fucosylated Gal3Man3GlcNAc6 (2231.7930 m/z and 2377.8509 m/z, respectively) are upregulated with therapy-resistance (Supplementary Fig. 15) and were among the glycans demonstrating significant magnitude of change in SCNC (Fig. 8b and Supplementary Fig. 16). Thus, we explored the ability of the level of these glycans to predict NE differentiation. Our results show that loss of non-fucosylated and fucosylated Gal3Man3GlcNAc6 have excellent predictive power for NE tumor regions (AUC = 0.9149) (Fig. 8e, f), which we confirm with mixed effects statistical analysis (*p* = 1.81 × 10^−4^ and 1.45 × 10^−5^, respectively). This indicates these structures could be useful markers for monitoring whether patients with therapy-resistant PCa are developing CRPC-NE.

Our results suggest that NE differentiation is associated with downregulation of complex N-glycans with the levels of high-mannose glycans remaining largely maintained between the two regions. We acknowledge that the higher proportion of NE tumor regions relative to adenocarcinoma regions in this cohort is a potential factor limiting the depth and conclusions of this study.

## Discussion

Aberrant glycosylation is a hallmark of cancer that has remained largely unexplored in many cancer settings^10^, including PCa. Despite the fact that there have been many studies that have sought to explore the molecular features of advanced, therapy-resistant PCa^5,7–9^, very few have studied glycans. We anticipate that this has historically been due to technological limitations and lack of tissue resources. The recent development of N-glycan IMS by the Drake Lab^26^ has allowed us the ability to quantify glycan abundance in pure tumor regions across a variety of tissue-types representing the major disease states of PCa, enabling effective modeling, for the first time, of glycosylation changes throughout the evolution of PCa.

Our results demonstrate that there is a high degree of interpatient variability in the early stage of PCa with very few glycans demonstrating consistent quantitative change across all patients. Tumors treated with hormonal therapy demonstrate a strong upregulation of complex biantennary structures with a decrease in tri- and tetraantennary structures. However, as resistance develops, the tri- and tetraantennary forms become aberrantly upregulated, indicating that these glycans are associated with disease activity. The functional consequences of this increased branching of glycans with the development of therapy resistance have yet to be determined but represents a critically important question. Our analysis shows that most of the triantennary structures associated with active disease contain bisecting GlcNAc while most of the tetraantennary glycans lack sialic acid. Furthermore, there is strong upregulation of higher molecular weight high mannose structures in CRPC, particularly Man9, and Glc1Man9. In patients who develop SCNC, there is significant downregulation of complex N-glycans. As the luminal secretory cells, which comprise adenocarcinomas, are major producers of glycoproteins, this may suggest that NE cells produce less glycoproteins compared to the luminal-type cells. High mannose glycans are largely maintained between CRPC-adeno and SCNC indicating that this class of glycans may be important therapeutic targets for advanced, therapy-resistant PCa irrespective of histology. The reduced Glc1Man9 in NE tumor cells suggests that SCNC has less endoplasmic reticulum (ER) stress compared to adenocarcinoma tumor cells. However, this requires further study. Furthermore, it is important to note that uncommon histological subtypes of PCa have been reported, including double-negative (AR-, NE-), amphicrine, carcinoid, and squamous-type carcinomas. Further studies would be required to determine if the above findings are consistent in these rarer pathological contexts.

This study shows that the glycosylation profile of tumor cells demonstrate significant change as a result of hormonal therapy and the development of AR-independent PCa. Many of these glycans, as our results have shown, can have great diagnostic potential and the development of agents for feasibly detecting and targeting some of these structures would be a great initial step for bridging the fields of cancer biology and glycobiology. Furthermore, several structures have significant therapeutic potential due to their disease-specific presence (certain high mannose glycans, tetraantennary glycans with poly-LacNAc repeats, etc). N-glycans are only found on cell surface and secreted proteins^29^ making them potentially excellent molecules for both detecting tumor cells and their products as well as targeting them. Many of the agents currently available are antibodies targeting certain structural features rather than clearly defined glycans. For example, a strong interest in Lewis antigens by the broader scientific community has prompted the development of antibodies towards sialyl Lewis X, sialyl Lewis A, Lewis B, and Lewis Y^34^. Although not N-linked structures, there have been many agents developed targeting Tn, sTn and T antigen^34^. Despite being potentially useful, specificity remains a major concern hindering the translation of these agents to the bedside. Recent improvements in the chemical synthesis of defined glycan structures (reviewed in ref. ^35^) has the potential to allow for the development of anti-glycan antibodies with higher specificity for cancer-associated structures. Advancing knowledge of the glycan structures associated with the progression of PCa and therapy resistance will allow significant progress towards treating patients beyond currently modalities to ultimately improve patient survival.

## Materials and methods

### Study design

The objective of this study was to survey changes in N-linked glycosylation across the common evolution of PCa. Unstained male human tissue slides, including whole slides and tissue microarray (TMA) slides, were used for IMS. Slides were then counterstained with hematoxylin & eosin (H&E), digitally scanned, and tumor regions were annotated using Aperio ImageScope. Glycan abundance within tumor regions were then analyzed and in vitro studies were carried out as needed to support major findings.

### FFPE tissues: whole slides and TMA

All human tissues used were originally obtained for diagnosis and/or treatment purpose. The de-identified residual archival tissue used in this study was approved by Duke University’s Institutional Review Board (IRB). A waiver of consent for the use of human tissues was provided from the IRB for this study because the tissues used were de-identified archival samples. All slides were randomly chosen and researchers were blinded to any demographic data. Hormone-sensitive PCa tissues consisted of a TMA representing 75 patients (150 cores) as well as 5 whole slide cases. Hormonally-treated PCa tissue consisted of a TMA representing 37 patients (97 cores). Hormone-resistant (refractory) PCa tissue consisted of 5 whole slide cases. Histologically heterogeneous hormone-sensitive PCa tissue (having both well-differentiated and low-differentiated glands on the same slide) consisted of 4 whole slide cases while SCNC tissue consisted of 5 whole slide cases.

### Deparaffinization and rehydration

All tissue and TMA slides were heated at 60 °C for 1 h. After incubation, tissues were deparaffinized by washing twice in xylene (3 min each). Tissue sections were then rehydrated by submerging the slide twice in 100% ethanol (1 min each), once in 95% ethanol (1 min), and once in 70% ethanol (1 min), and twice in water (3 min each). Following rehydration, the slides underwent heat-induced epitope retrieval (HIER) in citraconic anhydride buffer (25 µL citraconic anhydride in 50 mL HPLC H_2_O, pH 3) for 20 min at 95 °C in a decloaking chamber. After cooling, the buffer was exchanged with water five times (pouring out half of the buffer and replacement with water) prior to replacing completely with water. The slides were then desiccated prior to enzymatic digestion.

### N-Glycan IMS

Using an HTX M5 automated TMSprayer system, 15 passes of PNGase F (0.1 µg/µl in HPLC H_2_O) were applied to the desiccated tissues at a rate of 25 µl/min with a velocity of 1200 and a 3 mm offset at 10 psi and a nozzle temperature of 45 °C. Following application of PNGase F, slides were incubated at 37 °C for 2 h in a humidified chamber to deglycosylate the tissues. After deglycosylation, 10 passes of α-cyano-4-hydroxycinnamic acid (CHCA) matrix (7 mg/ml in 50/50/0.1 ACN/H_2_O/TFA) were applied to the tissues using the aforementioned HTX TMSprayer at a rate of 0.1 ml/min with a velocity of 1300 and a 2.5 mm offset at 10 psi and an 80 °C nozzle temperature. Released glycan ions were detected using a timsTOF Flex QTOF mass spectrometer operating in positive mode equipped with a Smartbeam 3D laser (10 kHz). 300 laser shots per pixel with a 20 µm beam footprint and a 40 µm lateral step size were used to obtain high-resolution images. Following MS acquisition, the data was imported in to SCiLS Lab imaging software (2022b Pro) and normalized to the total ion count. Observed glycans were annotated by comparison to an in-house database of known N-glycan structures and masses generated using Glycoworkbench^36^ and GlycoMod (web.expasy.org/glycomod/). Representative putative structures were determined by combinations of accurate m/z, CID fragmentation patterns, prior characterizations by both MALDI-TOF and RP-LC-MS/MS and the known glycan biosynthesis pathways.

### IMS data analysis

Following IMS, all slides were stained with hematoxylin and eosin (H&E) at the Duke University Biorepository Precision Pathology Center using a BioCare Medical Intellipath autostainer. Slides were then digitally scanned, studied histologically, and tumor regions were annotated using Aperio ImageScope. Annotated histological images were co-registered with the SCiLS Lab and subsequently used to generate regions of interests (ROI) for each tumor. These ROIs were used to assess the average abundance of all detected glycans within each annotated tumor region.

### Cell culture

LNCaP, C4–2, 22RV1, and PC-3 cells were obtained from ATCC and cultured in RPMI 1640 medium (Gibco) supplemented with 10% fetal bovine serum (Corning) and 1% penicillin-streptomycin (p/s) (Gibco). CS2 cells were cultured in RPMI 1640 medium (Gibco) supplemented with 10% charcoal-stripped serum (Corning) and 1% p/s (Gibco). VCaP was cultured in DMEM medium (Thermo) supplemented with 10% fetal bovine serum (Corning) and 1% p/s (Gibco) and LAPC4 was cultured in IMDM medium (Thermo) supplemented with 10% fetal bovine serum (Corning) and 1% p/s (Gibco). For androgen ablation experiments, LNCaP cells were cultured in RPMI medium containing 10% charcoal-stripped serum +1% p/s for 7 and 14 days, respectively prior to harvesting for flow cytometry. For AR inhibition experiments, LNCaP cells were cultured in RPMI medium containing 10% fetal bovine serum + 1% p/s + 10 µM Enzalutamide (Selleckchem). LNCaP cells to be studied as a control for the Enzalutamide-treated group were cultured in RPMI medium containing 10% fetal bovine serum + 1% p/s + DMSO (equivalent amount). All cell lines were authenticated prior to use.

### Flow cytometry

1 × 10^6^ cells from each cell line were harvested, washed twice with PBS, re-suspended in staining buffer (PBS + 0.2% FBS) and blocked (FcR Blocking Reagent, Miltenyi Biotec). Cells were then incubated with PHA-L lectin (GlycoMatrix) at a concentration of 10 µg/mL for 30 min at 4 °C. Following incubation, cells were washed twice with PBS, and re-suspended in staining buffer. 30,000 events were recorded for each tube using a BD LSRII Cell Analyzer. All data was further processed using FlowJo software.

### Statistical analysis

To screen for important glycan biomarkers involved in PCa progression, therapy resistance, and NE differentiation, t-tests were used to compare between different groups. The family-wise significance level was maintained at 0.05 by adjusting the false discovery rate using the Benjamini–Yekutieli procedure (Bejamini, Krieger, and Yekutieli). Multi-level model (mixed-effects model) was used in R Studio to account for potential correlations among different tumor regions within each patient to validate select glycans that were significant by T-test.

### Reporting summary

Further information on research design is available in the Nature Research Reporting Summary linked to this article.

## Supplementary information

Supplementary Information

REPORTING SUMMARY

Supplementary Data 1

Supplementary Data 2

Supplementary Data 3

Supplementary Data 4

Supplementary Data 5

Supplementary Data 6

Supplementary Data 7

Supplementary Data 8

Supplementary Data 9

## Data Availability

All data generated or analyzed are included in the manuscript and/or supplementary files.
