# Supplementary material for: Rewiring of the N-Glycome with prostate cancer progression and therapy resistance
Source: NPJ Precis Oncol. 2023 Feb 24;7:22. doi: 10.1038/s41698-023-00363-2 (PMC9958128; doi:10.1038/s41698-023-00363-2)
Supplement: Supplementary file 1 — Supplementary Information [file 41698_2023_363_MOESM1_ESM.pdf]

Supplementary Information

Supplementary Figures

Supplementary Figure 1: Summary of the major classes of N-glycans and common structural modifications

Supplementary Figure 2: Representative H&E images of a patient case with both well-differentiated and poorly differentiated tumor cells on same FFPE slide.

Supplementary Figure 3: Analysis of N-glycosylation changes between well differentiated and poorly differentiated glands within each patient

Supplementary Figure 4: Representative glycan image demonstrating upregulation of a biantennary glycan (2101.7551 m/z) in hormonally-treated tumors (specific tumor regions demarcated in black)

Supplementary Figure 5: Representative glycan image demonstrating downregulation of a tetraantennary glycan (1891.6924 m/z) in hormonally-treated tumors (specific tumor regions demarcated in black)

Supplementary Figure 6. Significant *N*-glycans with a high degree of quantitative change in hormonally treated tumors

Supplementary Figure 7: Gating Strategy for PHA-L Flow Cytometric Assays

Supplementary Figure 8: Flow cytometric analysis of hormone-sensitive cell lines and hormone-refractory cell lines for PHA-L reactivity

Supplementary Figure 9: Flow cytometric analysis of LNCaP cells for PHA-L activity and RT-qPCR demonstrating expression level of N-glycan branching enzymes

Supplementary Figure 10: MGAT4 and MGAT5 mRNA levels in hormone resistant PCa patients

Supplementary Figure 11. Significant *N*-glycans with a high degree of quantitative change in hormone resistant tumors

Supplementary Figure 12: Representative H&E images of a patient case with both adenocarcinoma and neuroendocrine tumor cells on same FFPE slide.

Supplementary Figure 13: Scatter plots depicting high mannose (Man 5-9) structure abundance between all adenocarcinoma (n = 17) and NE regions (n = 38)

Supplementary Figure 14: Analysis of N-glycosylation changes in patient with a high degree of mixed adenocarcinoma/neuroendocrine histology

Supplementary Figure 15: Scatter plots depicting non-fucosylated and fucosylated Gal3Man3GlcNAc6 levels in hormone-sensitive (n = 120), hormonally treated (n = 37), and hormone resistant (n = 75) tumor regions

Supplementary Figure 16: Significant N-glycans with a high degree of quantitative change in NE tumor regions

Supplementary Datasets (All supplementary datasets supplied as individual Excel files)

Supplementary Data 1: N-Glycan Mass Spectrometric Data Comparing N-Glycan Abundance Between Well-Differentiated and Poorly Differentiated Tumor Regions

Supplementary Data 2: N-Glycan Mass Spectrometric Data Comparing N-Glycan Abundance Between Well-Differentiated and Poorly Differentiated Tumor Regions for Patient 1

Supplementary Data 3: N-Glycan Mass Spectrometric Data Comparing N-Glycan Abundance Between Well-Differentiated and Poorly Differentiated Tumor Regions for Patient 2

Supplementary Data 4: N-Glycan Mass Spectrometric Data Comparing N-Glycan Abundance Between Well-Differentiated and Poorly Differentiated Tumor Regions for Patient 3

Supplementary Data 5: N-Glycan Mass Spectrometric Data Comparing N-Glycan Abundance Between Well-Differentiated and Poorly Differentiated Tumor Regions for Patient 4

Supplementary Data 6: N-Glycan Mass Spectrometric Data Comparing N-Glycan Abundance Between Hormone-Sensitive and Hormonally-Treated Tumor Regions

Supplementary Data 7: N-Glycan Mass Spectrometric Data Comparing N-Glycan Abundance Between Hormonally-Treated and Hormone-Resistant Tumor Regions

Supplementary Data 8: N-Glycan Mass Spectrometric Data Comparing N-Glycan Abundance Between Neuroendocrine and Mixed Adenocarcinoma Tumor Regions

Supplementary Data 9: N-Glycan Mass Spectrometric Data Comparing N-Glycan Abundance Between Neuroendocrine and Mixed Adenocarcinoma Tumor Regions in Patient 1

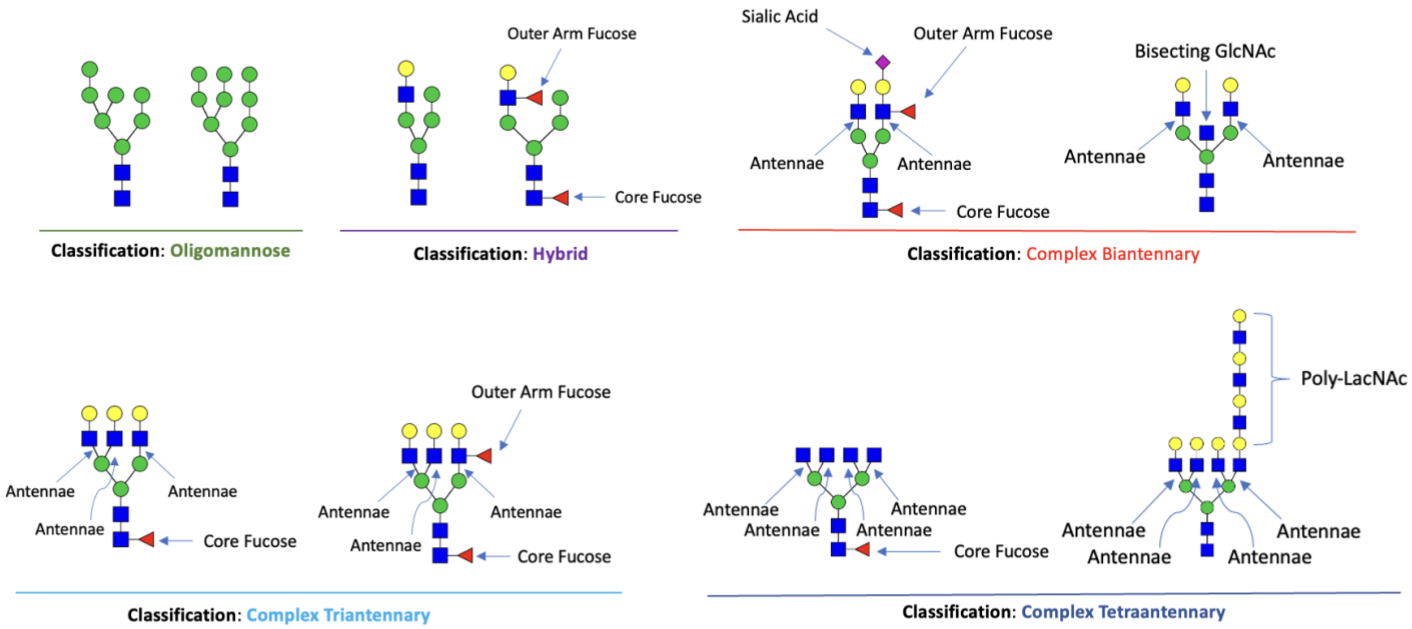

Supplementary Figure 1. Summary of the major classes of N-glycans and common structural modifications

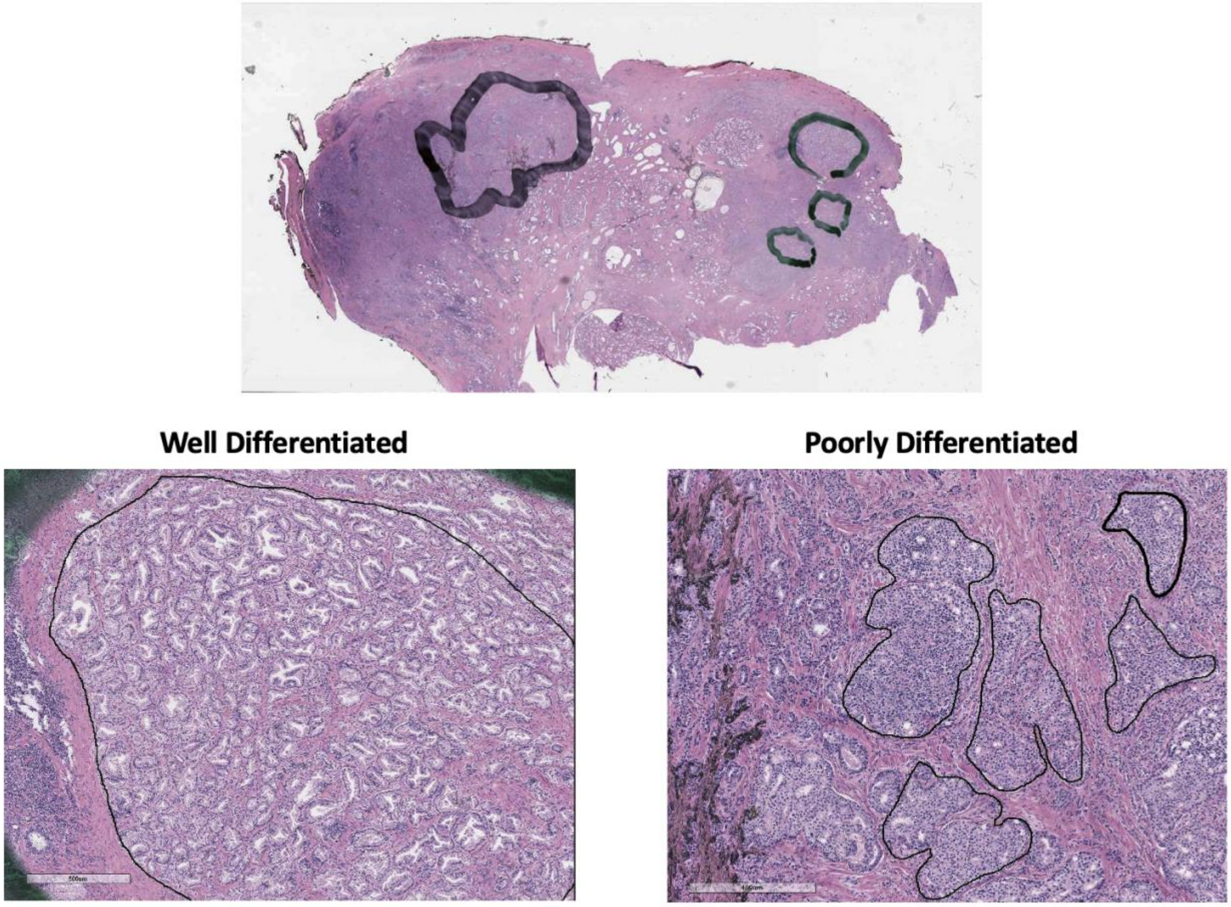

Supplementary Figure 2: **Representative H&E images of a patient case with both well-differentiated and poorly differentiated tumor cells on same FFPE slide. Upper panel demonstrates pathological annotation of targeted tumor regions (green = abundance of low grade tumor cells, black = abundance of high grade tumor cells). Lower panel demonstrates representative demarcated pure tumor cell regions within targeted areas.**

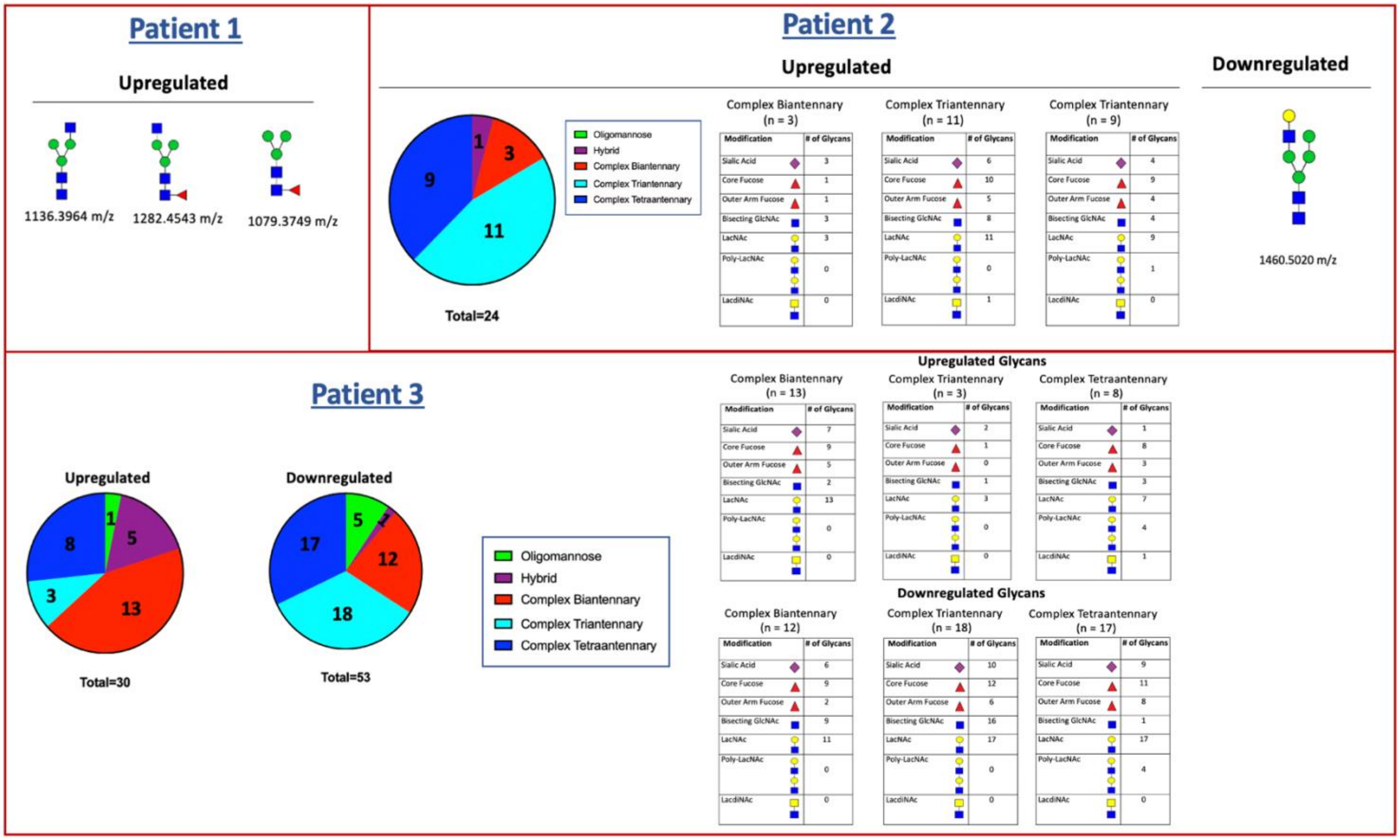

Supplementary Figure 3: Analysis of N-glycosylation changes between well differentiated and poorly differentiated glands within each patient. (Upper Left) Patient 1 demonstrates upregulation of 2 hybrid N-glycans (1136.3964 m/z and 1282.4543 m/z) and a core-fucosylated paucimannose structure (1079.3749 m/z) in poorly differentiated tumor regions (Upper Right) Patient 2 demonstrates upregulation of 24 structures and downregulation of 1 structure (1460.5020 m/z) in poorly differentiated tumor regions. The largest proportion of upregulated structures are complex tri- and tetraantennary forms. (Bottom) Patient 3 demonstrates upregulation of 30 structures and downregulation of 53 structures in poorly-differentiated tumor regions.. The largest proportion of upregulated structures were complex biantennary structures while the largest proportion of downregulated structures were complex tri- and tetraantennary forms.

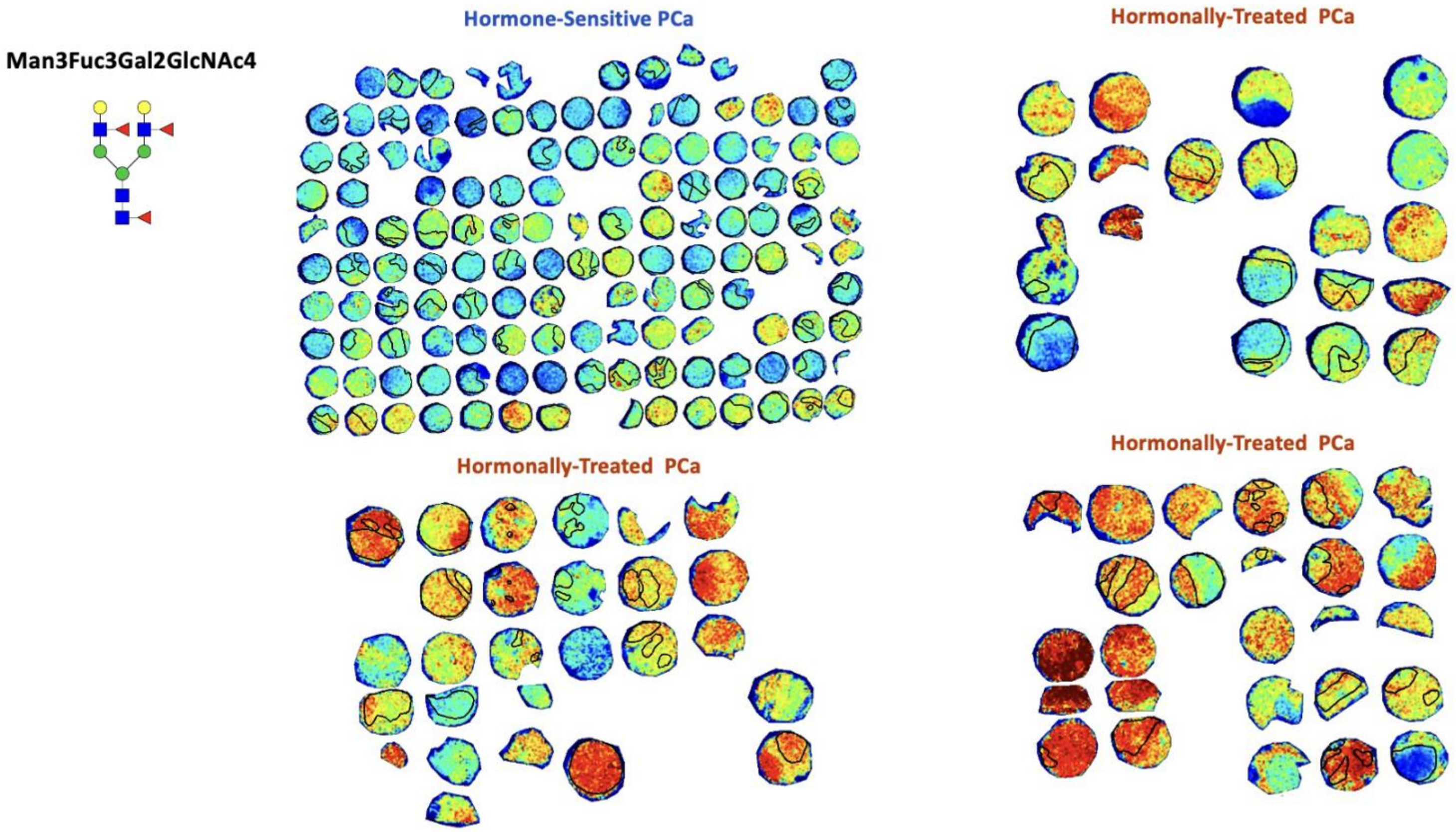

Supplementary Figure 4: Representative glycan image demonstrating upregulation of a biantennary glycan (2101.7551 m/z) in hormonally-treated tumors (specific tumor regions demarcated in black). Upregulated complex biantennary structures demonstrate localization both within demarcated tumor regions as well as in the surrounding stroma.

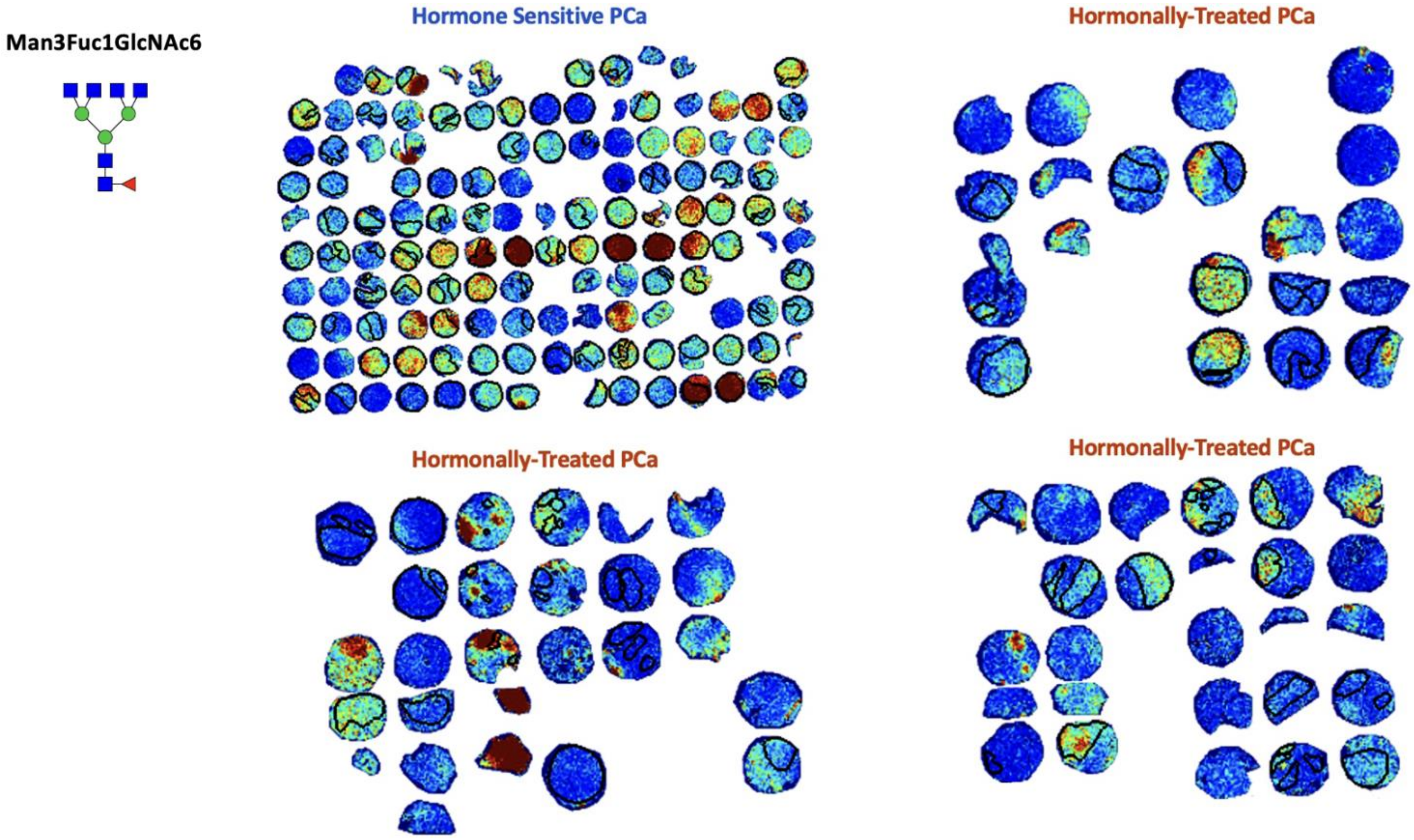

Supplementary Figure 5: Representative glycan image demonstrating downregulation of a tetraantennary glycan (1891.6924 m/z) in hormonally-treated tumors (specific tumor regions demarcated in black).

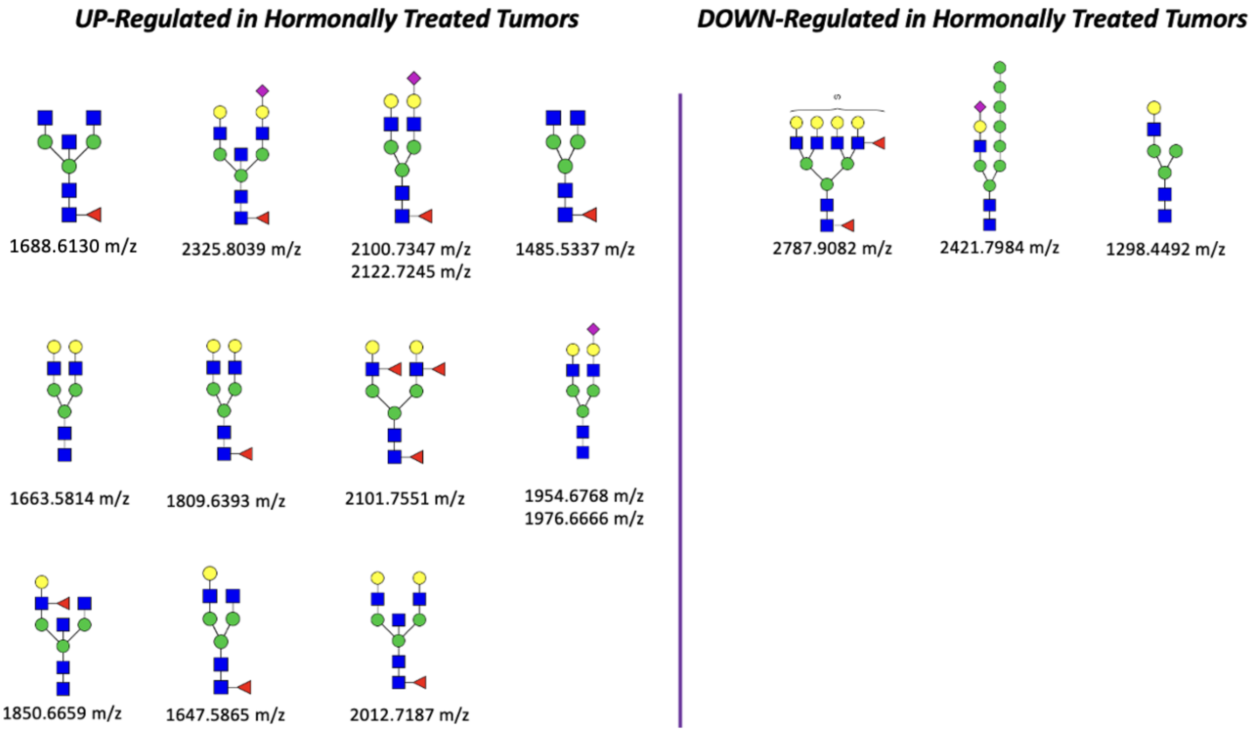

Supplementary Figure 6. Significant *N*-glycans with a high degree of quantitative change in hormonally treated tumors. These structure may serve as markers for PCa that is in remission and not yet resistant to androgen deprivation or AR inhibition.

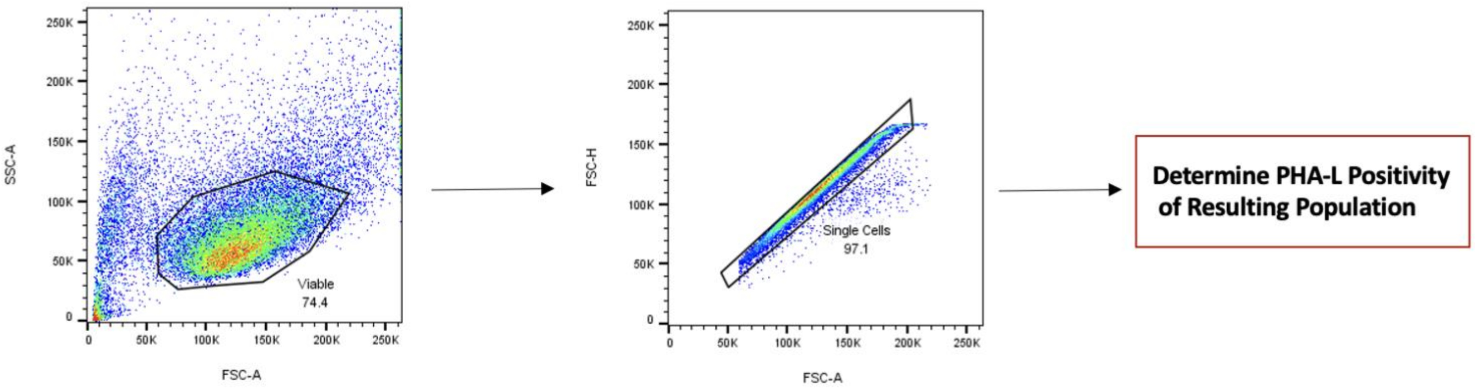

Supplementary Figure 7: Gating Strategy for PHA-L Flow Cytometric Assays

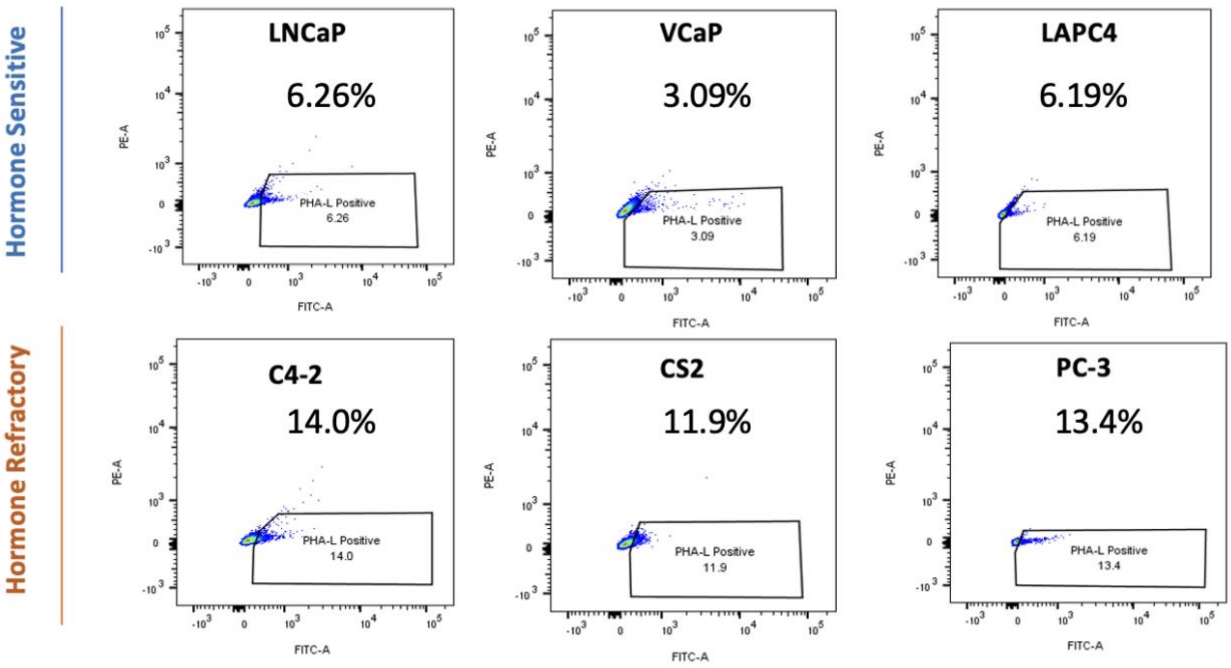

Supplementary Figure 8: Flow cytometric analysis of hormone-sensitive cell lines and hormone-refractory cell lines for PHA-L reactivity. PHA-L demonstrates the highest reactivity in hormone-refractory lines indicating these cells have a higher degree of β1-6 branched N-glycans on the cell surface.

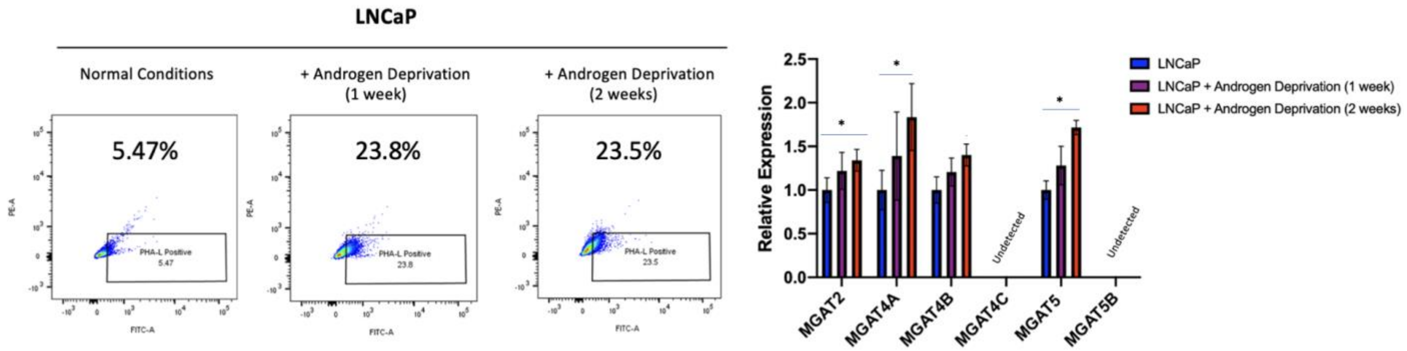

Supplementary Figure 9: Flow cytometric analysis of LNCaP cells for PHA-L activity and RT-qPCR demonstrating expression level of N-glycan branching enzymes. Androgen deprivation induces in vitro induces upregulation of branched N-glycans with elevated levels of MGAT2, MGAT4A, MGAT4B, and MGAT5. Two-way ANOVA was used for multiple comparisons with $p < 0.05$ considered significant. The following system was used to denote significance: $p < 0.05$ (*), $p < 0.01$ (**), $p < 0.0001$ (***), $p < 0.00001$ (****). Error bars correspond to standard deviation.

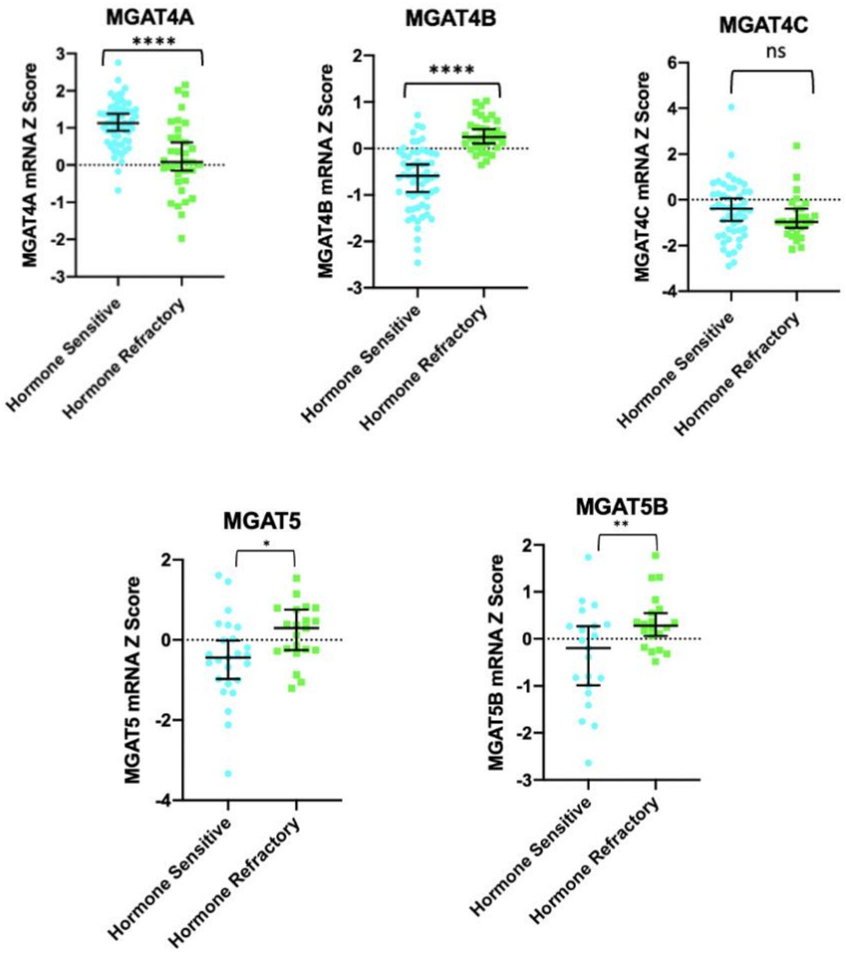

Supplementary Figure 10: MGAT4 and MGAT5 mRNA levels in hormone refractory PCa patients. MGAT4B, MGAT5, and MGAT5B demonstrate upregulated mRNA levels in hormone-refractory patients suggesting overall upregulated pathway activity. Statistics performed between each group by multiple, unpaired t test with $p < 0.05$ considered significant. The following system was used to denote significance: $p < 0.05$ (*), $p < 0.01$ (**), $p < 0.0001$ (***), $p < 0.00001$ (****). Error bars correspond to standard deviation.

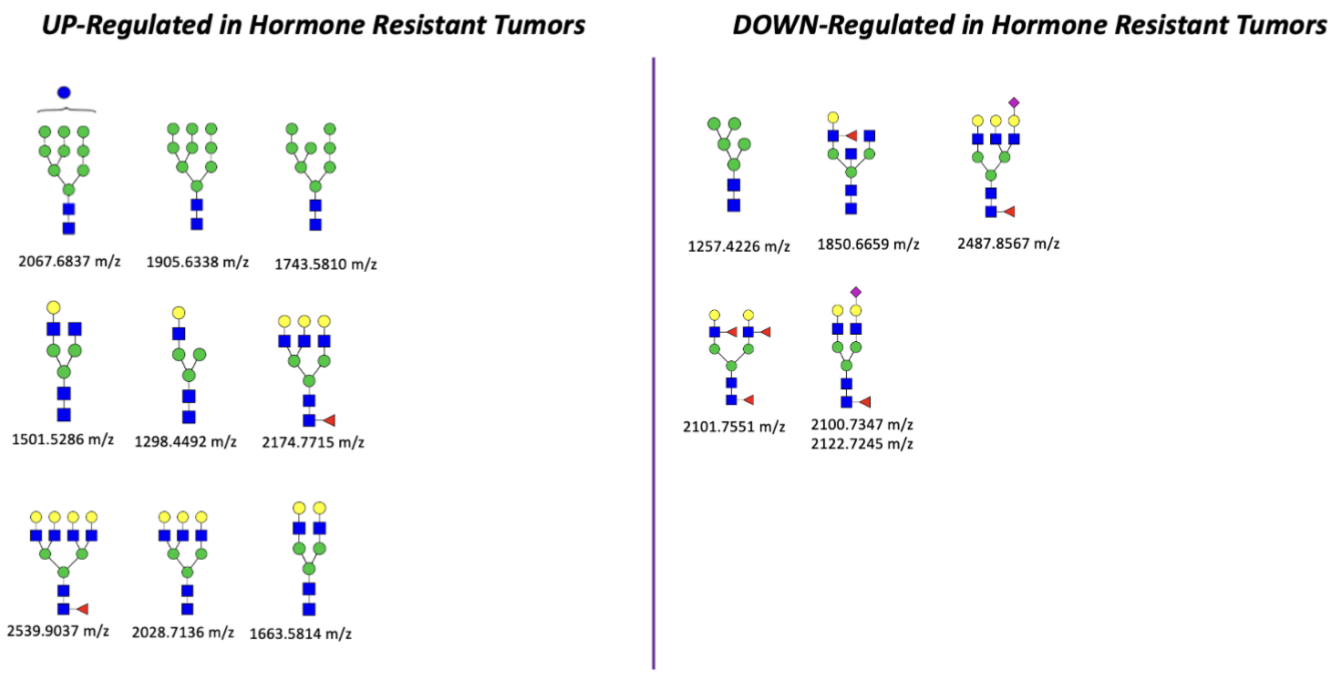

Supplementary Figure 11. Significant *N*-glycans with a high degree of quantitative change in hormone resistant tumors. These structures may serve as valuable markers for tumors that have developed resistance following hormonal therapy or be studied as potential therapeutic targets.

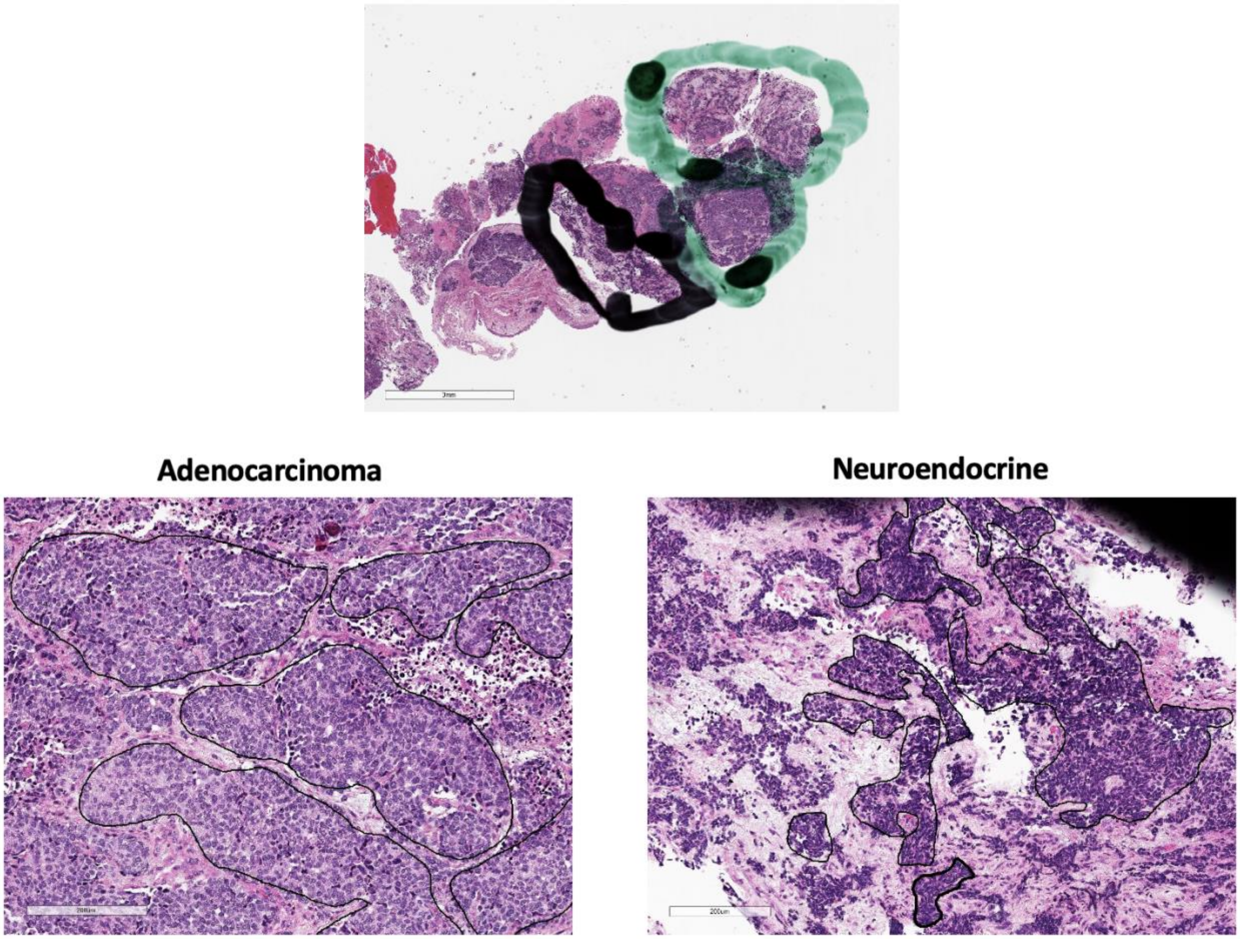

Supplementary Figure 12: Representative H&E images of a patient case with both adenocarcinoma and neuroendocrine tumor cells on same FFPE slide. Upper panel demonstrates pathological annotation of targeted tumor regions (green = abundance of adenocarcinoma tumor cells, black = abundance of neuroendocrine tumor cells). Lower panel demonstrates representative demarcated (black circles) pure tumor cell regions within targeted areas.

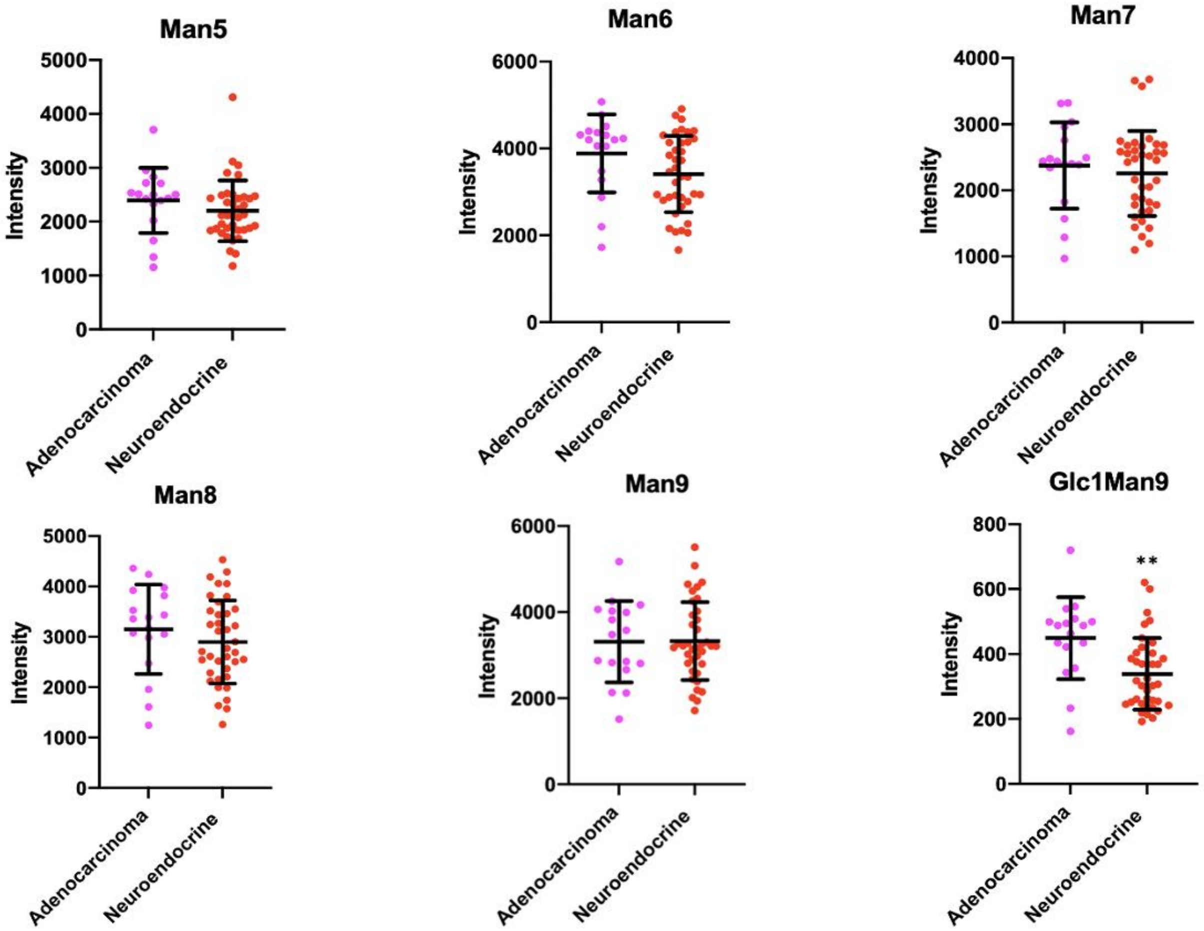

Supplementary Figure 13: Scatter plots depicting high mannose (Man 5-9) structure abundance between all adenocarcinoma (n = 17) and NE regions (n = 38). The levels of high mannose glycans remain relatively maintained between both regions with only Glc1Man9 demonstrating a small downregulation in NE tumor regions. Statistics performed between each group by multiple, unpaired t test with $p < 0.05$ considered significant. The following system was used to denote significance: $p < 0.05$ (*), $p < 0.01$ (**), $p < 0.0001$ (***), $p < 0.00001$ (****). Error bars correspond to standard deviation.

Intratumoral Analysis of Patient Displaying High Degree of Mixed Adenocarcinoma/Neuroendocrine Histology

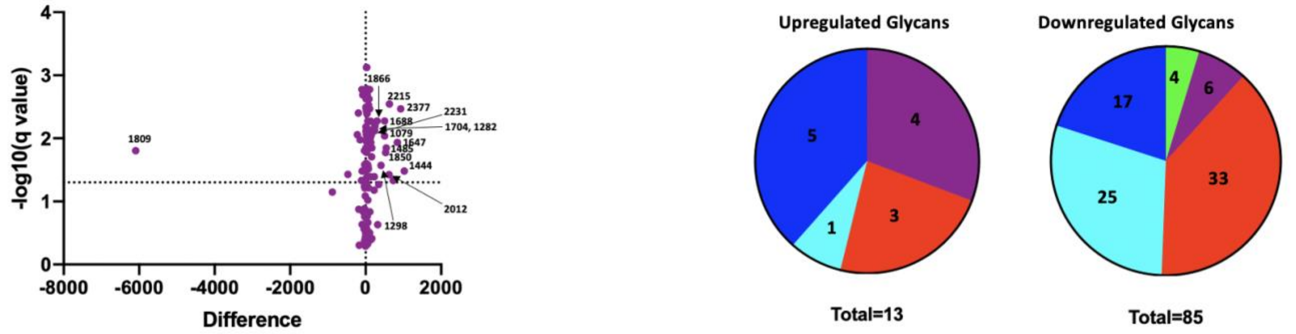

Upregulated

Complex Biantennary (n = 3)

Modification	# of Glycans
Sialic Acid	0
Core Fucose	3
Outer Arm Fucose	1
Bisecting GlcNAc	0
LacNAc	2
Poly-LacNAc	0
LacdiNAc	0

Complex Triantennary (n = 1)

Modification	# of Glycans
Sialic Acid	1
Core Fucose	0
Outer Arm Fucose	0
Bisecting GlcNAc	0
LacNAc	1
Poly-LacNAc	0
LacdiNAc	0

Complex Tetraantennary (n = 5)

Modification	# of Glycans
Sialic Acid	0
Core Fucose	5
Outer Arm Fucose	2
Bisecting GlcNAc	0
LacNAc	5
Poly-LacNAc	5
LacdiNAc	0

Downregulated

Complex Biantennary (n = 33)

Modification	# of Glycans
Sialic Acid	21
Core Fucose	21
Outer Arm Fucose	7
Bisecting GlcNAc	18
LacNAc	30
Poly-LacNAc	0
LacdiNAc	0

Complex Triantennary (n = 25)

Modification	# of Glycans
Sialic Acid	19
Core Fucose	17
Outer Arm Fucose	10
Bisecting GlcNAc	16
LacNAc	25
Poly-LacNAc	0
LacdiNAc	1

Complex Tetraantennary (n = 17)

Modification	# of Glycans
Sialic Acid	9
Core Fucose	13
Outer Arm Fucose	7
Bisecting GlcNAc	4
LacNAc	16
Poly-LacNAc	1
LacdiNAc	3

Supplementary Figure 14: Analysis of N-glycosylation changes in patient with a high degree of mixed adenocarcinoma/neuroendocrine histology. The vast majority of structures are downregulated and consist of complex-type glycans. Interestingly, in this patient, all upregulated tetraantennary glycans contained poly-LacNAc (a modification commonly associated with metastasis).

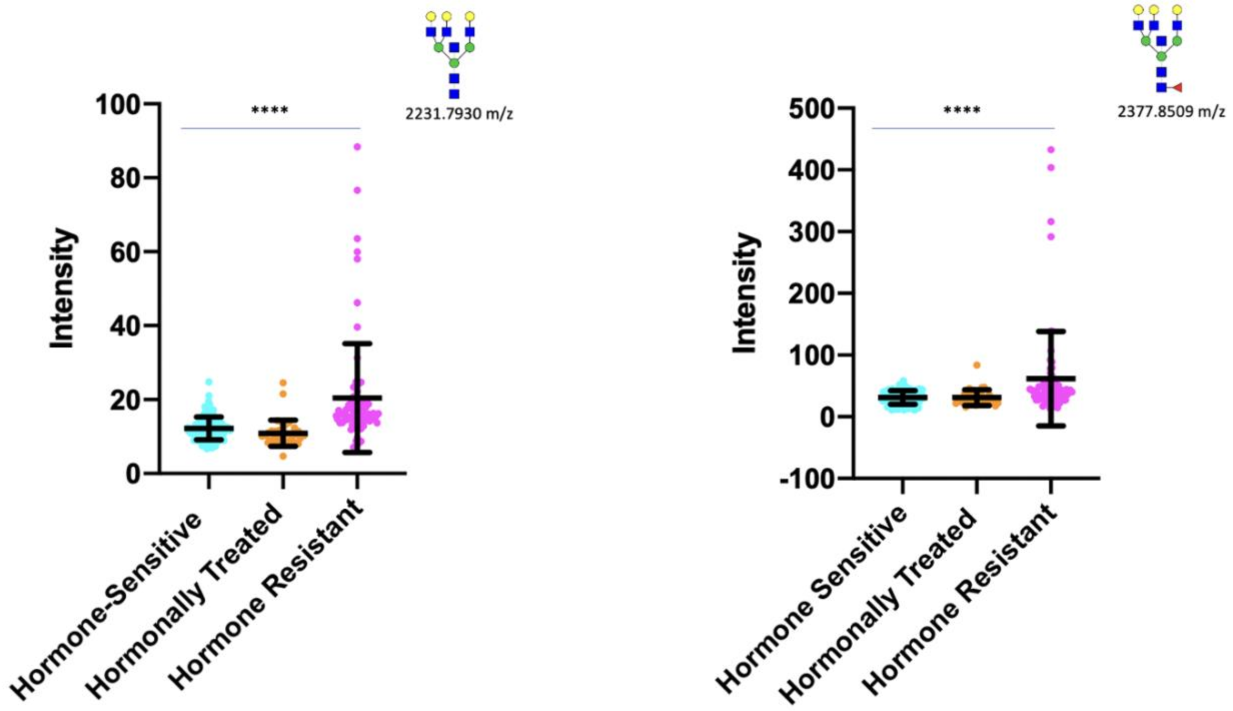

Supplementary Figure 15: Scatter plots depicting non-fucosylated and fucosylated Gal3Man3GlcNAc6 levels in hormone-sensitive (n = 120), hormonally treated (n = 37), and hormone resistant (n = 75) tumor regions. One-way ANOVA was used for multiple comparisons with $p < 0.05$ considered significant. The following system was used to denote significance: $p < 0.05$ (*), $p < 0.01$ (**), $p < 0.0001$ (***), $p < 0.00001$ (****). Error bars correspond to standard deviation.

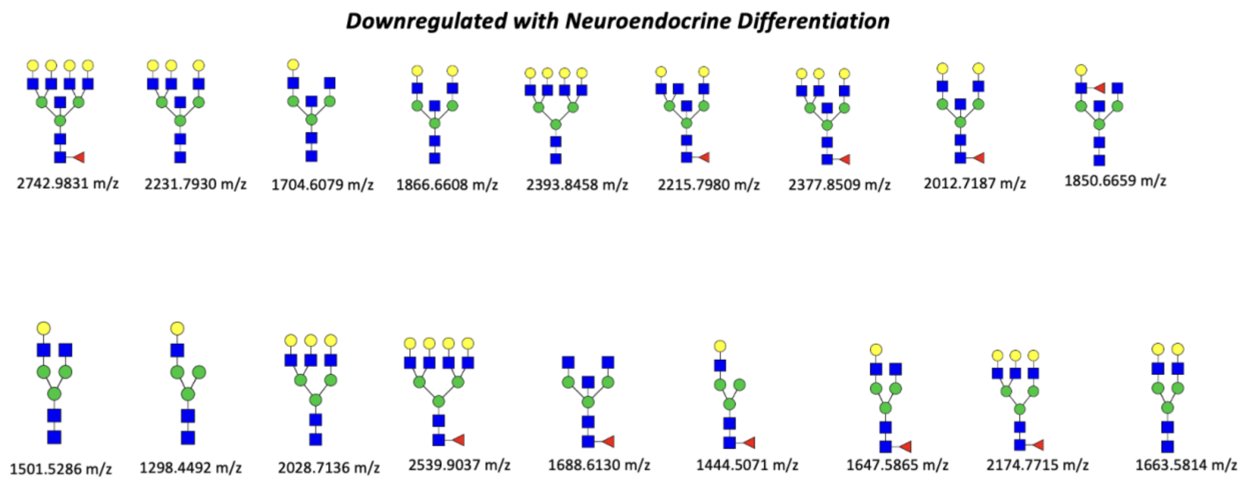

Supplementary Figure 16: Significant N-glycans with a high degree of quantitative change in NE tumor regions. These structures may serve as useful markers for determining whether tumors are undergoing NE differentiation.
