# Supplementary material for: Rewiring of the N-Glycome with prostate cancer progression and therapy resistance
Source: NPJ Precis Oncol. 2023 Feb 24;7:22. doi: 10.1038/s41698-023-00363-2 (PMC9958128; doi:10.1038/s41698-023-00363-2)
Supplement: Supplementary file 2 — REPORTING SUMMARY [file 41698_2023_363_MOESM2_ESM.pdf]

nature research

Corresponding author(s): Jiaoti Huang

Reporting Summary

Nature Research wishes to improve the reproducibility of the work that we publish. This form provides structure for consistency and transparency in reporting. For further information on Nature Research policies, see Authors & Referees and the Editorial Policy Checklist.

Statistical parameters

When statistical analyses are reported, confirm that the following items are present in the relevant location (e.g. figure legend, table legend, main text, or Methods section).

n/a	Confirmed	
☐	☒	The exact sample size (n) for each experimental group/condition, given as a discrete number and unit of measurement
☐	☒	An indication of whether measurements were taken from distinct samples or whether the same sample was measured repeatedly
☐	☒	The statistical test(s) used AND whether they are one- or two-sided<br>*Only common tests should be described solely by name; describe more complex techniques in the Methods section.*
☐	☒	A description of all covariates tested
☐	☒	A description of any assumptions or corrections, such as tests of normality and adjustment for multiple comparisons
☐	☒	A full description of the statistics including central tendency (e.g. means) or other basic estimates (e.g. regression coefficient) AND variation (e.g. standard deviation) or associated estimates of uncertainty (e.g. confidence intervals)
☐	☒	For null hypothesis testing, the test statistic (e.g. F, t, r) with confidence intervals, effect sizes, degrees of freedom and P value noted<br>*Give P values as exact values whenever suitable.*
☒	☐	For Bayesian analysis, information on the choice of priors and Markov chain Monte Carlo settings
☒	☐	For hierarchical and complex designs, identification of the appropriate level for tests and full reporting of outcomes
☐	☒	Estimates of effect sizes (e.g. Cohen's d, Pearson's r), indicating how they were calculated
☐	☒	Clearly defined error bars<br>*State explicitly what error bars represent (e.g. SD, SE, CI)*

Our web collection on statistics for biologists may be useful.

Software and code

Policy information about availability of computer code

Data collection	No software was used.
Data analysis	Statistical comparisons of all glycan abundances within different annotated regions were performed through Prism9, using the Two-stage step-up method (Benjamini, Krieger, and Yekutieli) with false discovery rate (FDR) of 5%. Multi-level model (mixed-effects model) was used in R Studio to account for potential correlations among different tumor regions within each patient to validate select glycans that were significant by T-test.

For manuscripts utilizing custom algorithms or software that are central to the research but not yet described in published literature, software must be made available to editors/reviewers upon request. We strongly encourage code deposition in a community repository (e.g. GitHub). See the Nature Research guidelines for submitting code & software for further information.

Data

Policy information about availability of data

All manuscripts must include a data availability statement. This statement should provide the following information, where applicable:

- Accession codes, unique identifiers, or web links for publicly available datasets
- A list of figures that have associated raw data
- A description of any restrictions on data availability

The data that supports the findings of this study are available from the corresponding authors upon reasonable request.

Field-specific reporting

Please select the best fit for your research. If you are not sure, read the appropriate sections before making your selection.

☒ Life sciences ☐ Behavioural & social sciences ☐ Ecological, evolutionary & environmental sciences

For a reference copy of the document with all sections, see nature.com/authors/policies/ReportingSummary-flat.pdf

Life sciences study design

All studies must disclose on these points even when the disclosure is negative.

Sample size	The number of patient cases for each group was dictated based on the # of available tissue samples in Pathology since this study involved rarely resected tissue. A statistician (co-author on this paper) was heavily involved to make sure the number of samples per group would generate valid conclusions from the data by T-test. In experiments with lower number of samples per group (< 5 samples), a mixed effects model was used to control for patient variability in the validation of select glycan markers.
Data exclusions	No data was excluded from the analysis.
Replication	All analyses involved multiple patients. Major findings central to the conclusions of the paper were validated in vitro.
Randomization	Due to the nature of the study, samples were allocated into groups based on disease stage and treatment status (i.e. hormone-sensitive, hormonally-treated, CRPC-adenocarcinoma, CRPC-neuroendocrine).
Blinding	Investigators were blinded to group allocation during analyses.

Reporting for specific materials, systems and methods

Materials & experimental systems

n/a	Involved in the study	
☒	☐	Unique biological materials
☐	☒	Antibodies
☐	☒	Eukaryotic cell lines
☒	☐	Palaeontology
☒	☐	Animals and other organisms
☒	☐	Human research participants

Methods

n/a	Involved in the study	
☒	☐	ChIP-seq
☐	☒	Flow cytometry
☒	☐	MRI-based neuroimaging

Antibodies

Antibodies used	Phaseolus vulgaris Lectin (PHA-L) - FITC, GlycoMatrix, SKU #: 30330033-1, Lot #: L21012703DVAK
Validation	The lectin was validated by the manufacturer with certificate of analysis and relevant citations available on the GlycoMatrix website. Furthermore, since MGAT5 induces formation of the product of PHA-L, we have tested the lectin in MGAT5-overexpressing cells which further confirmed the ability of PHA-L to detect complex, branched glycans.

Eukaryotic cell lines

Policy information about cell lines

Cell line source(s)	ATCC
Authentication	All cell lines used were obtained from ATCC which follows rigorous authentication procedures (sterility testing, CO1, and STR profiling)
Mycoplasma contamination	All cell lines used tested negative for mycoplasma contamination.
Commonly misidentified lines (See ICLAC register)	*Name any commonly misidentified cell lines used in the study and provide a rationale for their use.*

Flow Cytometry

Plots

Confirm that:

☒ The axis labels state the marker and fluorochrome used (e.g. CD4-FITC).

☒ The axis scales are clearly visible. Include numbers along axes only for bottom left plot of group (a 'group' is an analysis of identical markers).

☒ All plots are contour plots with outliers or pseudocolor plots.

☒ A numerical value for number of cells or percentage (with statistics) is provided.

Methodology

Sample preparation	1x106 cells from each cell line (obtained from ATCC) were harvested, washed twice with PBS, re-suspended in staining buffer (PBS + 0.2% FBS) and blocked (FcR Blocking Reagent, Miltenyi Biotec). Cells were then incubated with PHA-L lectin (GlycoMatrix) at a concentration of 10 ug/mL for 30 minutes at 4 °C. Following incubation, cells were washed twice with PBS, and re-suspended in staining buffer. 30,000 events were recorded for each tube using a BD LSRII Cell Analyzer. All data was further processed using FlowJo software.
Instrument	BD LSRII Cell Analyzer
Software	DiVa was used for collecting data on the BD LSRII Cell Analyzer. FlowJo software was used for subsequent analysis.
Cell population abundance	Cells were not sorted. Flow cytometry was only used to determine the
Gating strategy	FSC/SSC gating followed by FSC-H/FSC-A gating was used to select only single, viable cells for analysis. An unstained control was subsequently used for determining the FITC+ population which corresponded to cells binding PHA-L.

☒ Tick this box to confirm that a figure exemplifying the gating strategy is provided in the Supplementary Information.
